# Elucidating the Genetic Architecture of Fiber Quality in Hemp (*Cannabis sativa* L.) Using a Genome-Wide Association Study

**DOI:** 10.3389/fgene.2020.566314

**Published:** 2020-09-17

**Authors:** Jordi Petit, Elma M. J. Salentijn, Maria-João Paulo, Christel Denneboom, Eibertus N. van Loo, Luisa M. Trindade

**Affiliations:** ^1^Wageningen UR Plant Breeding, Wageningen University & Research, Wageningen, Netherlands; ^2^Biometris, Wageningen University & Research, Wageningen, Netherlands

**Keywords:** GWAS, *Cannabis sativa*, hemp, fiber quality, cell wall composition, RAD-sequencing, QTL

## Abstract

Hemp (*Cannabis sativa* L.) is a bast-fiber crop with a great potential in the emerging bio-based economy. Yet, hemp breeding for fiber quality is restricted and that is mainly due to the limited knowledge of the genetic architecture of its fiber quality. A panel of 123 hemp accessions, with large phenotypic variability, was used to study the genetic basis of seven cell wall and bast fiber traits relevant to fiber quality. These traits showed large genetic variance components and high values of broad sense heritability in this hemp panel, as concluded from the phenotypic evaluation across three test locations with contrasting environments. The hemp panel was genotyped using restriction site associated DNA sequencing (RAD-seq). Subsequently, a large set (> 600,000) of selected genome-wide single nucleotide polymorphism (SNP) markers was used for a genome-wide association study (GWAS) approach to get insights into quantitative trait loci (QTLs) controlling fiber quality traits. In absence of a complete hemp genome sequence, identification of QTLs was based on the following characteristics: (i) association level to traits, (ii) fraction of explained trait variance, (iii) collinearity between QTLs, and (iv) detection across different environments. Using this approach, 16 QTLs were identified across locations for different fiber quality traits, including contents of glucose, glucuronic acid, mannose, xylose, lignin, and bast fiber content. Among them, six were found across the three environments. The genetic markers composing the QTLs that are common across locations are valuable tools to develop novel genotypes of hemp with improved fiber quality. Underneath the QTLs, 12 candidate genes were identified which are likely to be involved in the biosynthesis and modification of monosaccharides, polysaccharides, and lignin. These candidate genes were suggested to play an important role in determining fiber quality in hemp. This study provides new insights into the genetic architecture of fiber traits, identifies QTLs and candidate genes that form the basis for molecular breeding for high fiber quality hemp cultivars.

## Introduction

Hemp (*Cannabis sativa* L.) is a bast-fiber crop with a great potential in the emergent bio-based economy. It is an environmentally friendly crop that fits well into crop rotation scenarios and sustainable agriculture. Besides, it can also be used for bio-remediation purposes of polluted lands (Struik et al., 2000; Amaducci et al., 2008; van den Broeck et al., 2008; Amaducci and Gusovius, 2010). This multi-purpose crop is, apart from being a valuable source of cannabinoids and oils (Salentijn et al., 2015), an alternative and more sustainable source of fibers relative to water and nutrient demanding crops, (e.g., cotton) and to non-renewable glass and fossil-based fibers (Ebskamp, 2002; van der Werf and Turunen, 2008; Amaducci and Gusovius, 2010). Despite the large interest in hemp, it is a relatively poorly developed crop. This is mostly a consequence of the strong decline in hemp production in the last century, when intensive breeding programs drove great improvements and amplified cultivation of major staple crops (Salentijn et al., 2015). Subsequently, hemp breeding was restricted and to date little is known about the genetic architecture that underlies hemp fiber quality.

Recently, large phenotypic variability in 28 traits related to fiber quality of hemp has been reported in a diverse panel of 123 hemp accessions native to different regions in the world (Petit et al., 2020). This study, conducted in trials in three European locations, reported that fiber quality in hemp is a clear example of quantitative trait, in which cumulative effects of genetic and environmental factors, as well as genotype and environment interactions (*G* × *E*), play important roles. Yet, the mode of regulation of the different traits was found to differ, ranging from traits with large genetic components, large heritabilities, and low *G* × *E* interactions to traits that are largely controlled by environmental components, with small contributions of genetic factors.

Fibers of high quality for different purposes and easily extractable from the stems are among the traits with the greatest appreciation by the hemp industry. More specifically, these include high bast fiber content, high content of cellulose, and low content of pectin in the cell walls, fine bast fibers and efficient decortication properties of the stems (Ranalli, 2004; Salentijn et al., 2015). The association of lignin content and fiber quality depends on their applications. Briefly, low lignin content in the bast fibers is associated to high quality of fiber for textile purposes. The lignin polymers hinder decortication and increases the stiffness of the fibers (Ranalli, 2004). On the other hand, the antioxidant properties of lignin and its adhesion functions, which are associated to increase the stability in the composites, boost the interest of lignin for innovative applications of the fibers (reviewed in Pickering et al., 2016). The contents of bast fiber, cellulose and lignin were reported to be largely determined by genetic components in the hemp accession panel from Petit et al. (2020), suggesting that the panel is a promising dataset to further study the genetic basis of these traits. In contrast, the variation of pectin, bast fiber fineness and decortication efficiency were largely controlled by environmental factors, which would hamper the study of their genetic components. For these low heritable traits, Petit et al. (2020) suggested agronomic practices, as they showed adaptive behavior under certain environmental conditions.

Despite the large variability of hemp fiber quality and the large influence of genetics on important traits of fiber quality, the genetic mechanisms controlling these traits remain mostly unknown in hemp. Different genetic approaches can be performed to study its genetic basis, such as reverse genetics (candidate gene approaches) and forward genetics (genetic mapping studies). Salentijn et al. (2019) reviewed the state of art of candidate gene studies for fiber quality in hemp. Briefly, most of the reported genes have a function in the lignin metabolism or code for phytohormones involved in plant development with a possible effect on lignin (Salentijn et al., 2019). However, many different metabolic pathways are involved in the biosynthesis and regulation of the cell walls. As a consequence, the function of an altered gene might be replaced by another one, whereby the genes are functionally redundant (Boerjan et al., 2003; Pauly et al., 2013; Kumar et al., 2016). To date, only three genetic association studies have been performed in hemp and they focused on sex expression and cannabinoid content (Faux et al., 2016; Grassa et al., 2018; Laverty et al., 2019). These studies were based on biparental mapping population approaches to detect quantitative trait loci (QTLs) in a genetic map. Despite the interest, to the best of our knowledge, no association studies for fiber quality of hemp and no genome-wide association studies (GWAS) have been reported for hemp. The lack of a complete genome sequence (Sawler et al., 2015; Laverty et al., 2019) and the lack of panels of hemp accessions harboring large variability in fiber quality have hampered such studies on this crop (Sawler et al., 2015). As a result, no QTLs have been reported for hemp fiber quality. Furthermore, hemp breeding programs are currently based on conventional breeding, while molecular breeding has not yet been developed in hemp (Salentijn et al., 2015). Molecular breeding would accelerate the development of new cultivars with improved fiber properties. This approach would allow the selection of promising individuals at early developmental stages, reducing the time and costs of breeding programs. Genetic association studies are thus of great value to upgrade breeding programs toward molecular approaches.

Insights in the genetic architecture of hemp fiber quality are essential to develop new breeding strategies, which seek for novel hemp cultivars with improved fiber properties. The objective of this study was to identify QTLs associated to fiber quality. The highly variable panel of 123 diploid hemp accessions (2*n* = 20) was used for this purpose (Petit et al., 2020). GWAS analyses were performed for fiber quality traits, with large genetic components. The study was performed in three test locations with contrasting environments.

## Materials and Methods

### Plant Material

A panel of 123 hemp accessions was used in this study (Supplementary Table 1; Petit et al., 2020). The panel included mostly fiber type accessions but also an oil seed cultivar, landraces and breeding material. The panel originated from different countries in Europe, Asia (China), and North America (Canada). Plants were cultivated in three locations across Europe to assess different environments: Rovigo (CRA – Centro di ricerca cerealicoltura e colture industriale, Italy, 45°N 11°E), Chèvrenolles, Neuville-sur-Sarthe (FNPC – Fédération Nationale des Producteurs de Chanvre, France, 48°N 0.2°E), and Westerlee (VDS – Vandinter Semo BV, Netherlands, 53°N 6°E). The multi-location trial was established between April and September of 2013. Each location had a randomized complete block design with three biological replicates per accession. The experimental units were plots of 1 m^2^ in Italy and the Netherlands and of 1.5 m^2^ in France.

### Phenotyping and Data Collection

Phenotyping of cell wall traits was performed essentially as described in Petit et al. (2019) and in Petit et al. (2020). Briefly, six cell wall traits [contents of glucose (Glc%dm), glucuronic acid (GlcA%dm), mannose (Man%dm), xylose (Xyl%dm), acid detergent lignin (ADL%dm), and Klason lignin (KL%dm)] and one fiber parameter (BCD%) were measured after harvesting the stems. Plants were cut from the based (10 cm above the ground) when the accumulative temperature degree days (Σ°C, the accumulated Celsius degree day over a period at a base temperature of 1°C) were 1740.25Σ°C, 1421.1Σ°C, and 1843.3Σ°C in CRA, FNPC, and VDS, respectively. These temperatures corresponded to the Σ°C when most accessions reached full flowering in each location. The biochemical composition of the cell walls from stems of the 123 accessions was measured with multivariate prediction models based on near-infrared spectroscopy (NIRS). Data were predicted for each accession in each block and in each location. The bast fiber content after decortication (BCD%) was measured as an average of 10 stems per plot after warm water retting and decortication, using a lab-scale roller-breaker decortication system, according to Wang et al. (2018) and Petit et al. (2020).

### DNA Extraction

Genomic DNA was isolated from young grinded hemp leaves (∼20–400 mg, lyophilized material) using a cetyl trimethyl ammonium bromide (CTAB) method (Doyle and Doyle, 1987) with additional steps to remove proteins, polysaccharides, and RNA. First, the leave material was treated with proteinase K in 500 μl TES buffer (100 mM TRIS pH = 8.0, 10 mM EDTA pH = 8.0, 2% SDS, 200 μg/ml proteinase K) for 1 h at 60^*o*^C. Thereafter, the CTAB method was performed using a final concentration of 1% CTAB and 1.4 N NaCl. Polysaccharides were removed by incubation for 1 h on ice in 1.2 M NH4Ac, centrifugation for 10 min at 13,000 rpm and discarding the pellet. The supernatant was then treated with RNAseA (100 μg RNAseA per 700 μl supernatant, 30 min at 37°C) and extracted with chloroform: isoamyl alcohol (IAA). The DNA was precipitated and dissolved in 550 μl ultra-pure water. The CTAB method was repeated on this sample and the resulting DNA was suspended in 30 μl ultra-pure water. DNA samples were further purified over a column (Genomic DNA Clean and concentrator-10, Zymo Research) and controlled for quality and DNA concentration on agarose gel and by Qubit^^TM^ Fluorometric quantitation to provide high quality genomic DNA for massive sequencing.

Hemp is an outcrossing species and each accession used in the GWAS panel might have a certain degree of genetic heterogeneity, despite being phenotypically homogeneous (Sawler et al., 2015). Therefore, to cover all allelic variation within accessions, the genomic DNA of eight individual plants per accession was isolated and pooled in equimolar amounts, resulting in 123 samples for genotyping by restriction site associated DNA sequencing (RAD-seq).

### Restriction-Site-Associated DNA Sequencing (RAD-Seq)

The GWAS mapping panel was sequenced with RAD-seq to identify single nucleotide polymorphisms (SNPs) distributed over the genome to be used as molecular markers. High quality genomic DNA (2.5–5 μg at a concentration ≥ 25 ng/μl) was digested using the restriction enzyme *Eco*RI. Then, RAD libraries with insert sizes of 300–550 bp, were prepared for each sample, as described by Baird et al. (2008). The 123 samples were paired end sequenced on an Illumina platform (PE150) in two rounds to provide 2 × 1 Gbp genomic data per sample. RAD library preparation and sequencing were performed by Beijing Genomics Institute (BGI, Hong Kong).

### RAD-Seq Data Analysis

Adaptors from the sequences were trimmed and low quality reads were removed. Low quality reads comprised reads with > 50% of the bases Q ≤ 12, unknown bases > 3%, reads that lacks a part of the multiplexing barcode, and could not be identified, and reads lacking the key sequence of the enzyme used. The clean sequence reads of each sample were mapped to the *C. sativa* ‘Purple Kush’ (canSat3 version GCA_000230575.1) genome reference (van Bakel et al., 2011), using Burrows–Wheeler Alignment Tool (Li and Durbin, 2009; specific BWA parameters: (o) max number or fraction of gap opens = 1; (e) max number or fraction of gap extensions = 50; (m) maximum entries in the queue = 100,000). Picard-tools (v1.118) was used to sort the Sequence Alignment Map (SAM) files by coordinate and convert them to Binary Alignment Map (BAM) files and to mark duplicate reads. The average mapping rate was 55.54% (range 50.3–85.7%). Subsequently, SOAPsnp was used to call SNPs in each sample (Li et al., 2009).

### SNP Marker Selection

Samples for each accession consisted of pools of eight diploid plants to cover all allelic variation present in the accessions. Each sample therefore harbors DNA from 16 alleles, represented by mostly expected two different nucleotides but occasionally three and rarely four different nucleotides (A,G,C, and T) at a position. For each polymorphic site, the possible allele frequencies (%A, %G, %C, and %T) were calculated per accession and in the GWAS panel.

Quality SNP marker selection was performed based on a 100% call rate of the SNPs in the 123 hemp accessions. Markers with a minor allele frequency below 2% and with a major allele frequency above 98% in the mapping panel were removed. Only biallelic markers were selected, the frequency sum of the two major alleles was equal or above 95%. In addition, to ensure allelic variation in the mapping panel, the markers with a standard deviation in the frequency of the major allele below 0.1 were removed. In total a set of 612,452 SNPs was selected for the genetic analysis. Each SNP was scored as the proportion of the major allele in the pool sample of plants from the same accession. Quality SNP marker selection was performed in R version 3.4.3 statistical software.

### Analysis of Population Structure

A kinship matrix was used to study the genetic relatedness between the accessions. The VanRaden (2008) method, following the same approach as in Kruijer et al. (2015), was used to calculate the kinship matrix by using the entire set of selected markers. To investigate the patterns of population structure, a principal coordinate analysis (PCoA) of the kinship matrix was performed using Genstat 19th edition. A dendrogram of the kinship was also calculated in R version 3.4.3 statistical software, using *APE* package (Paradis et al., 2004). In addition, clusters of accessions inferred from the kinship matrix were further analyzed to study the level of population structure in the GWAS panel. Pairwise comparisons of coefficient of population differentiation (*F*_ST_) between clusters were calculated using the Wright’s formula (Wright, 1969):

(1) $$F_{\text{ST}}=\frac{\bar{p}\,\left(1-\bar{p}\right)-\sum c_{\text{i}}\,p_{\text{i}}\,\left(1-p_{\text{i}}\right)}{\bar{p}\,\left(1-\bar{p}\right)}=\frac{\bar{p}\,\left(1-\bar{p}\right)-p\,\left(1-p\right)}{\bar{p}\,\left(1-\bar{p}\right)}$$

where the allele frequency in the *i*th population is *p*_i_, the relative size of the *i*th population is *c*_i_, and the average allele frequency between the two populations is $\bar{p}$.

### Analysis of Pairwise Correlation Between Markers

The identification of the boundaries of QTLs with a fragmented genome sequence in scaffolds is difficult. The study of the pairwise correlation between markers in certain genomic regions and by mapping groups of collinear markers (MultiQTL modeling, see section “Genome-Wide Association Study (GWAS) Analysis”) can overcome this limitation.

**FIGURE 1:**
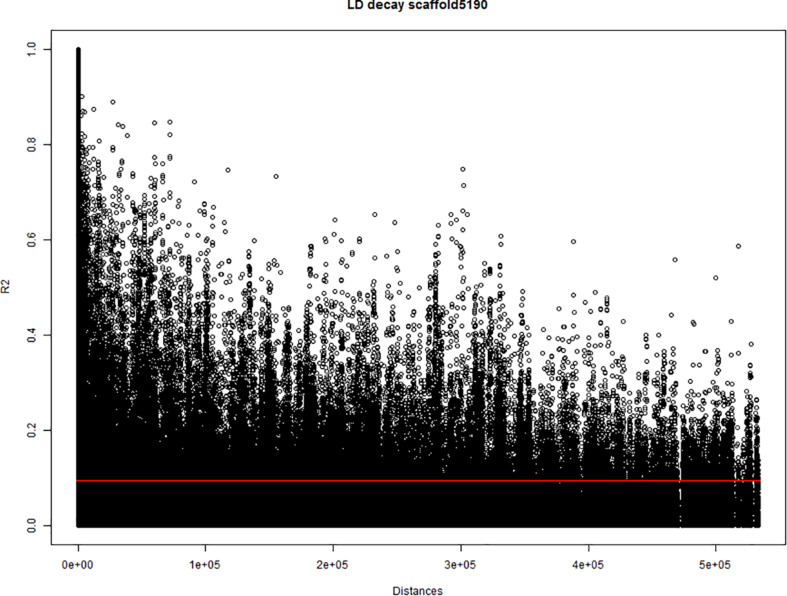
Estimation of the linkage disequilibrium (LD) decay of the largest scaffold (scaffold5190; 565.9 kbp) covered with single nucleotide polymorphism (SNP) markers. The *X*-axis indicates the physical distances and the *Y*-axis indicates the pairwise correlations (*r*^2^) between all pairs of markers from the scaffold. Red line indicates the critical value of *r*^2^.

To estimate the threshold to discriminate between collinear and non-collinear markers, a study of pairwise correlation-distance between markers from the same scaffold was performed. The study was performed on the 24 largest scaffolds (∼130–∼600 kbp) of the cannabis genome canSat3 version GCA_000230575.1 (van Bakel et al., 2011), that were covered with SNP markers. The analysis used the marker allele frequencies of the 123 hemp accessions to calculate the squared-allele frequency correlation (*r*^2^) between pairs of bi-allelic markers. The correlation values (*r*^2^) were compared with the physical distance between the marker pairs. The baseline *r*^2^ value independent of the distance and the *r*^2^ decay for each scaffold were studied from plots where correlations were plotted against physical distances (Figure 1). Correlation analyses were performed using Genstat 19th edition and plots were generated in R version 3.4.3 statistical software.

In most tested genomic scaffolds, the *r*^2^ decayed with the distance. Nevertheless, a baseline value of *r*^2^ close to 0.1 was detected in all scaffolds independently of the distance between markers. This baseline value of *r*^2^ (< 0.1) was set as a threshold to identify non-random associated markers. When markers non-affected by population structure and were correlated with r^2^ equal or above 0.1 (∼*r* ≥ 0.3), they were hypothesized to be physically close on the same chromosome. Therefore, *r*^2^ ≥ 0.1 was used as threshold for collinearity of markers in the multiQTL models (see section “Genome-Wide Association Study (GWAS) Analysis”). For those markers involved in genetic relatedness processes, genetic drift and selection can create linkages between markers, even if they are located in different chromosomes. The correlation study between marker frequencies also detected large correlations between markers that are far apart (distances up to ∼500 kbp and correlations up to *r*^2^ = 0.6). This indicated that a fraction of the QTLs can be located in alleles that comprise non-random correlation between markers over long physical distances. Thus, QTLs can be composed of different genomic scaffolds.

### Genome-Wide Association Study (GWAS) Analysis

A Linear Mixed Model (LMM) was used to identify significant associations between SNP markers and fiber quality traits, using a kinship correction (VanRaden, 2008) and following the same approach as in Yu et al. (2006), Huang et al. (2010), Malosetti et al. (2011), Zhou and Stephens (2012), and Kruijer et al. (2015). Briefly, the effects of the SNP markers on the phenotypic variation were studied with the fixed effects of the model. The kinship was used to control for population structure effects and was set in the random effects of the model. The kinship was calculated using all selected markers (∼600 k), following the equations to calculate the genomic relationship matrix developed by VanRaden (2008). LMM analyses were performed using restricted maximum likelihood (REML) algorithm and the kinship was calculated using R^1^ version 3.4.3 statistical software. The linear mixed model equation for our association with kinship correction is expressed as:

(2) γ=X⁢α+K⁢β+e

Equation (2) is a standard linear mixed model in which γ represents the phenotype, *X* is the marker, α is the effect size of the marker, *K* is the kinship for population structure correction, β is the effect of the population structure and ***e*** is the residual effects. *X*α represents the fixed effects, and *K*β, and *e* the random effects. Wald statistic from the REML analysis was used to assess the significant level of the associations. To account for multiple testing and estimate the threshold for significant associations, we performed a Bonferroni correction based on the number of independent markers (Li and Ji, 2005). The cumulative distributions of observed and expected *p*-values were inspected for 3,000 randomly selected SNP markers, to assess the correction for population structure (Yu et al., 2006). The expected *p*-values were the significance level of the associations between genotypes and phenotypes under the assumptions that markers were unlinked to the polymorphism controlling the variation of the traits. An independent analysis was performed separately for seven fiber quality traits of hemp and over three locations.

A MultiQTL model was performed following a forward selection procedure to identify QTLs associated to a trait. Here, a QTL is defined as a group of significant and collinear QTL-markers, represented by the marker that explains the largest phenotypic variance (representative QTL-marker). The MultiQTL model is the combination of non-collinear QTLs, whereby each QTL explains a specific part of the phenotypic variation in the population. First, the best representative QTL-marker was selected into the model. To avoid multicollinearity in the MultiQTL models, correlations between the selected marker and remaining candidate markers were determined. All significant markers correlated at *r* ≥ 0.3 (∼*r*^2^ ≥ 0.1) were considered collinear to the first one. The remaining significant markers (*r* < 0.3) were considered candidates to add to the model and forward selection was continued with those. The percentage of the total phenotypic variation explained by the genetic effects of the full MultiQTL model was calculated by the correlation (*r*^2^) between the fitted trait values and the observed traits values. The explained variance is an approximation to the heritability of the trait.

The same value of threshold for collinearity described in section 2.8 was confirmed using a different approach. Different conditions of correlation (*r* value) were used in the MultiQTL models, ranging from *r* = 0.1 to *r* = 0.9. At a threshold of *r* ≤ 0.3 (∼*r*^2^ ≤ 0.1) the number of QTLs and explained variance remained mostly steady, while at *r* > 0.3 (∼*r*^2^ > 0.1) the number of QTLs and the explained variance dramatically increased (Supplementary Table 2).

In order to find groups of significant markers, a principal component analysis (PCA) was performed. The PC1, PC2, and the –log_10_*P* of the association between genotypes and phenotypes were plotted in 3D scatter plots to detect the clusters. The 3D scatter plots resembled the peak tops of the QTLs in Manhattan plot distributions.

To further characterize the genetics of fiber quality traits across locations, a correlation analysis was performed between the QTLs of the three MultiQTL models for each trait, corresponding to the three test locations. For a trait, a common QTL region can be composed of different genomic scaffolds, that harbor correlated (*r* ≥ 0.3; *r*^2^ ≥ 0.1) representative QTL-markers from MultiQTL models of two or three locations.

Genome-wide association study (GWAS), cumulative distribution analyses, PCA and correlation analyses were performed in Genstat 19th edition software (VSN International, Hemel Hempstead, United Kingdom). The 3D scatter plots were performed in Excel version 14.0, using the macro Excel 3D Scatter Plot version 2.1 (Doka, 2013).

### Transcriptome Annotation and Candidate Gene Identification

The transcriptome of the hemp cultivar *C. sativa* ‘Finola’ (finola1), aligned to the *C. sativa* ‘Purple Kush’ (canSat3 version GAC 000230575.1) assembly, was downloaded from the *C. sativa* Genome Browser Gateway at http://genome.ccbr.utoronto.ca/cgi-bin/hgGateway (van Bakel et al., 2011). The transcriptome of ‘Finola’ was annotated using Blast2go (blastx 2.8.0; *E* value cut of 0.001; Conesa et al., 2005; Conesa and Gotz, 2008). The annotated transcriptomic data are deposited in the 4TU.ResearchData archive^2^. Genomic scaffolds associated to the QTLs were analyzed for transcripts. Special focus was given to genes with predicted functions related to the biosynthesis and modification of the cell wall, based on gene description and relevant research papers.

The sequences of the candidate genes were blasted using Blast + (Camacho et al., 2009) to the transcriptome BioProject PRJNA435671 (Behr et al., 2019) to identify the corresponding *Arabidopsis* homologues.

## Results

### Development of Markers for Genome-Wide Association Studies

Restriction site associated DNA sequencing generated 3,717.57 million clean reads (557.4 Gb) with an average of 29.7 million reads per sample (range 15.1–49.2 millions) with an average read length of 149.9 bp. Our genotyping approach resulted in the detection of 2,852,901 SNPs with a missing rate < 50% in the population (number of samples with missing data divide by total number of samples). These SNPs were used for genotyping by scoring nucleotide frequencies in each accession. In total 612,452 SNPs were selected as informative markers for the genetic analysis. Selected markers covered 35,590 scaffolds of the draft canSat3 genome (van Bakel et al., 2011), including the largest scaffolds. In total, a cumulative length of ∼503 Mb was covered by the scaffolds, which corresponds to ∼59.7% of the haploid genome sequence of hemp (843 Mb).

### Low Level of Population Structure in the Hemp Panel

**FIGURE 2:**
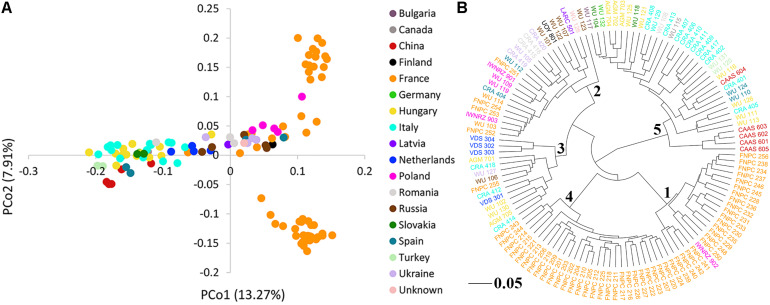
Population structure of the 123 hemp accessions. **(A)** Principal coordinates analysis (PCoA) and **(B)** dendrogram of the kinship matrix with the number of the sub-cluster in the node. Color palette indicates the geographic origin of the accessions.

The PCoA of the kinship matrix revealed the presence of some structure among the 123 hemp accessions. PCo1 divided most French accessions from the others with some levels of admixture and PCo2 divided French accessions in two groups. Nonetheless, PCo1 and PCo2 only explained 23.18% of the total genetic variance (Figure 2A). The dendrogram of the kinship matrix revealed a similar structure (Figure 2B). Five groups were identified, three of them showed large level of admixture, including accessions from different origin, whereas the remaining two clusters virtually showed only French accessions. The coefficients of population differentiation (*F*_ST_) between the five groups showed low levels of differentiation between them, ranging from 0.019 to 0.058 (Table 1).

**TABLE 1:** Pairwise coefficients of population differentiation (*F*_ST_) between the five clusters of the genome-wide association study (GWAS) panel inferred from the dendrogram from the kinship matrix (Figure 1).

Clusters	1	2	3	4	5
1	–				
2	0.034296	-			
3	0.05169	0.019437	–		
4	0.04746	0.033938	0.043606	–	
5	0.058453	0.034621	0.015365	0.054827	–

### Effective Control of Population Structure in Highly Heritable Fiber Quality Traits

The extensive phenotypic variation and the large genetic components of several cell wall traits (contents of glucose, mannose, xylose, glucuronic acid, Klason lignin, and acid detergent lignin) and bast fiber content make these traits excellent candidates to study the genetic architecture of fiber quality of hemp (Supplementary Tables 3, 4). The high heritabilities of these seven traits allow the study of the additive control of fiber quality traits. In addition, their interactions between genotypes and environments (*G* × *E*) allow to study the heritable variation in fiber quality sensitive to the environment (Petit et al., 2020).

**FIGURE 3:**
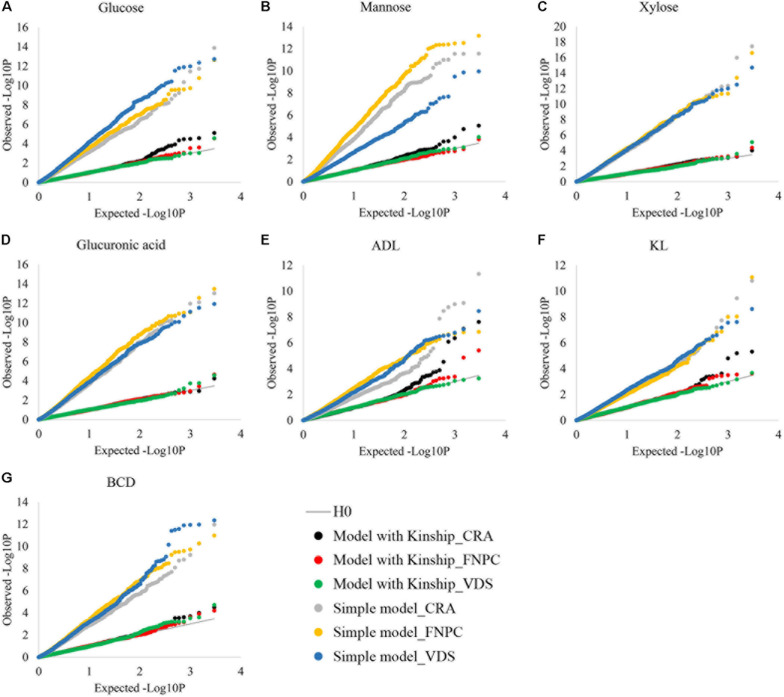
Cumulative distributions of *p*-values for contents of glucose **(A)**, mannose **(B)**, xylose **(C)**, glucuronic acid **(D)**, acid detergent lignin (ADL; **E)**, Klason lignin (KL**; F)** and Bast fiber content after decortication (BCD**; G)** to assess the correction for population structure. *X*-axes = the cumulative distributions of the expected-log_10_*P* for the simple and the kinship models; *Y*-axes = the cumulative distributions of the observed-log_10_*P* for the simple and the kinship models; Simple models = models without correction for population structure {dots in gray [Centro di ricerca cerealicoltura e colture industriale (CRA = Italy)], yellow [Fédération Nationale des Producteurs de Chanvre (FNPC) = France], and blue [Vandinter Semo BV (VDS) = The Netherlands]}; Kinship models = models with correction for population structure [dots in black (CRA = Italy), red (FNPC = France), and green (VDS = The Netherlands)]; H0 = models under the expectation that random single nucleotide polymorphism (SNP) markers are unlinked to the polymorphism controlling the traits (gray line).

To control putative effects of population structure in the phenotypic variation of highly heritable traits, the efficiency of the kinship matrix was assessed in the seven fiber quality traits, as depicted in Figure 3. Without the kinship matrix, observed –log_10_*P* values were higher than the expected ones, suggesting large number of false-positive associations. In contrast, when the effects of population structure were controlled by the kinship matrix, observed and expected –log_10_*P* values were similar for the seven fiber quality traits. These results show that the kinship matrix efficiently controlled the effects of population structure on the seven traits, which is key to identify true-positive associations. The phenotypic data of the seven traits can therefore be used to get insights into the genetic control of fiber quality.

### Significant Associations Between Markers and Fiber Quality Traits in Hemp

The GWAS analyses resulted in the identification of over 2,500 markers, mapping to single loci on 1,515 different genomic scaffolds. These associations were found significant (-log_10_*P* ≥ 4.047) for at least one trait and one location (Supplementary File 1). These results indicated that the number of significant markers in each genomic scaffold was mostly one to three markers per scaffold. Yet, some scaffolds showed higher number of significant markers, such as scaffold4465 (length of 14,465 bp) that showed 12 significant markers (Supplementary File 1). Moreover, the distribution of significant markers within the scaffolds showed differences between different scaffolds. For instance, the 12 significant markers from scaffold4465 were spread in a genomic sequence of ∼9,000 bp, while the 11 significant markers from scaffold34707 clustered in a shorter sequence of ∼600 bp. Moreover, markers that clustered close to one edge of the scaffold sequence (i.e., significant markers from scaffold4465) indicated that the putative flanking genomic sequence of that scaffold could also have significant markers associated to the same trait/s. Furthermore, several markers were significantly associated to several fiber quality traits. For example, many markers from scaffold4465 were significantly associated to contents of glucose, xylose, lignin (ADL, KL) and bast fiber content (Supplementary File 1). These results suggest a common genetic control between different traits.

### Identification of QTLs for Trait and Location: MultiQTL Models

Altogether, 90 QTLs were detected in the models among all traits and locations. The number of QTLs detected for the same trait generally differed across locations. The explained variance of the models for the same traits also differed across locations. In total, only eight of the QTLs were detected in more than one location for the same trait or were detected common in different traits (Table 2). These differences were likely to be explained by the significant *G* × *E* interactions on the traits (Supplementary Table 4).

**TABLE 2:** Quantitative trait loci (QTLs) for fiber and cell wall traits of hemp.

Trait	Type of Trait	Location	QTLs (*n*)	Explained Variance	Representative QTL-Markers
ADL%dm	Cell wall	CRA	3	60.32	scaffold73277_3647; scaffold74027_10718; scaffold4782_157368
ADL%dm	Cell wall	FNPC	5	40.2	scaffold34707_3473; scaffold80551_6348; scaffold2608_892; scaffold46943_424; scaffold60230_6140
ADL%dm	Cell wall	VDS	4	56.15	scaffold12000_76415; scaffold4268_1985; scaffold12558_4900
Glc%dm	Cell wall	CRA	3	27.75	scaffold45492_8629; scaffold15962_51386; scaffold71896_5178
Glc%dm	Cell wall	FNPC	5	63.08	scaffold55006_23494; scaffold118257_4014; scaffold25092_982; scaffold137175_654; scaffold53823_15026
Glc%dm	Cell wall	VDS	3	55.78	scaffold69515_8050; scaffold18086_2629; scaffold8221_7904
GlcA%dm	Cell wall	CRA	13	60.87	scaffold82406_5533; scaffold66904_776; scaffold111279_1125; scaffold16765_1949; scaffold9389_35935; scaffold39478_12834; scaffold11938_20472; scaffold2003_8850; scaffold2733_12157; scaffold869_24280; C32032885_328; scaffold29101_9917; scaffold1838_41611
GlcA%dm	Cell wall	FNPC	3	47.19	scaffold90847_19970; scaffold15717_124591; scaffold76130_8395
GlcA%dm	Cell wall	VDS	6	57.23	scaffold55265_18829; scaffold68246_496; scaffold5954_52112; scaffold20722_6013; scaffold79841_7656; scaffold28137_3788
KL%dm	Cell wall	CRA	2	40.11	scaffold45492_8629; scaffold10943_380
KL%dm	Cell wall	FNPC	6	40.2	scaffold53823_15026; scaffold63415_1601; scaffold69322_5540; scaffold47933_3213; scaffold68105_6992; scaffold6550_126605
KL%dm	Cell wall	VDS	3	48.53	scaffold12000_76415; scaffold51533_356; scaffold13365_3919
Man%dm	Cell wall	CRA	6	39.93	scaffold17542_9887; scaffold2731_11599; scaffold9919_11591; scaffold47229_16597; C32103569_1584; scaffold17032_37572
Man%dm	Cell wall	FNPC	7	66.64	scaffold34580_21508; scaffold12140_37109; scaffold4854_60; scaffold71367_190; scaffold34081_8008; scaffold900_4618; scaffold48672_475
Man%dm	Cell wall	VDS	2	46.59	scaffold90847_19970; scaffold32213_3500
Xyl%dm	Cell wall	CRA	4	23.38	scaffold103588_1151; scaffold49748_10229; scaffold30777_901; scaffold14559_5718
Xyl%dm	Cell wall	FNPC	4	47.43	scaffold42215_3622; scaffold14590_17087; scaffold25801_1953; scaffold3268_34793
Xyl%dm	Cell wall	VDS	3	32.92	scaffold55265_18829; scaffold56299_8302; scaffold32213_3500
BCD	Fiber	CRA	3	41.8	scaffold56299_8302; scaffold13466_34599; scaffold35378_13209
BCD	Fiber	FNPC	3	61.08	scaffold26831_57993; scaffold25080_10706; scaffold62704_12566
BCD	Fiber	VDS	2	45.71	scaffold56299_8302; scaffold3696_33201

*Number of QTLs (*n*) identified in the respective MultiQTL models per trait and per test location (CRA = Italy; FNPC = France, and VDS = The Netherlands), with explained variance of the full model. Trait: ADL%dm, acid detergent lignin content; BCD, bast fiber content after decortication; Glc%dm, glucose content; GlcA%dm, glucuronic acid content; KL%dm, Klason lignin content; Man%dm, mannose content; and Xyl%dm, xylose content; Explained variance, percentage of the total phenotypic variation explained by the genetic effects of the full MultiQTL model. The explained variance is an approximation of the heritability contribution of the full model. Representative QTL-markers, QTL-markers that explain the largest variance in a MultiQTL model. Markers are indicated as the scaffold number and the position of the single nucleotide polymorphism (SNP) within the scaffold (canSat3 assembly GAC_000230575.1).*

**FIGURE 4:**
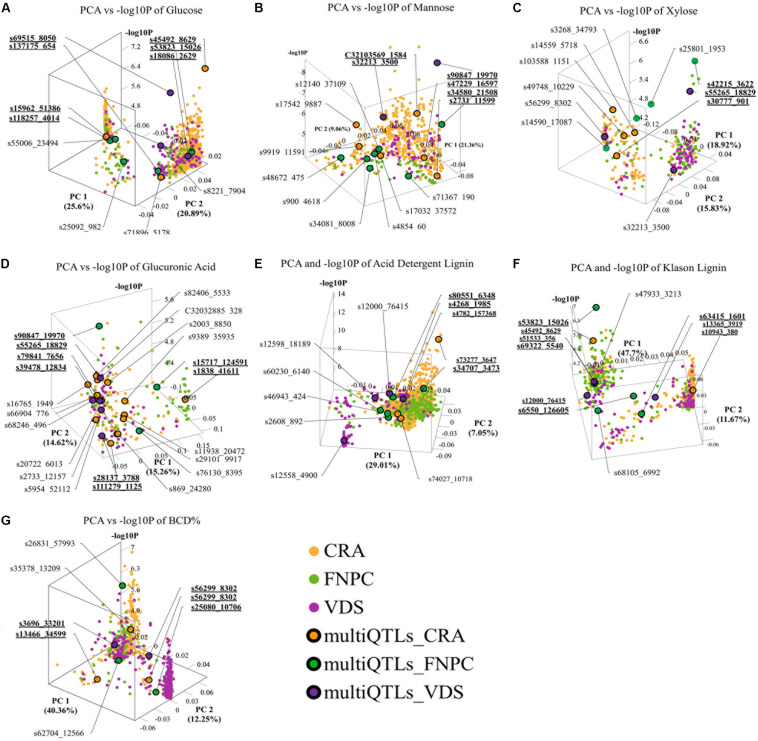
3D scatter plots of principal component analyses (PCAs), depicting variation in the allele frequency profiles of significant loci for respectively contents of glucose **(A)**, mannose **(B)**, xylose **(C)**, glucuronic acid **(D)**, acid detergent lignin (ADL; **E**), Klason lignin (KL; **F**), and bast fiber content after decortication (BCD; **G**). The plots resemble the peak tops of the quantitative trait loci (QTLs) in Manhattan plot distributions, despite the lack of physical positions on a chromosome. Each dot represents a significant QTL-marker (loci). *X*-axes = principal component 1 (PC1) with the percentage of explained variance; *Y*-axes = principal component 2 (PC2) with the percentage of explained variance; *Z*-axes = significant levels of the loci in the genome-wide association study (GWAS) association (-log_10_*P*); Orange, green, and purple dots = significant QTL-markers detected in respectively Centro di ricerca cerealicoltura e colture industriale (CRA), Fédération Nationale des Producteurs de Chanvre (FNPC), and Vandinter Semo BV (VDS); Orange, green, and purple dots with a black circle = representative QTL-markers of the MultiQTL models from respectively CRA, FNPC, and VDS; CRA = field trial in Italy; FNPC = field trial in France; VDS = field trial in Netherlands; QTL-names (e.g., s55006_23495) = QTL-markers from different MultiQTLs models; QTL-names in bold and underlined (e.g., **s69515_8050**) = QTL-markers from different MultiQTLs models that belong to a QTL region across locations. Each plot is shown in the angle that represents better the results. Principal Components and -log_10_*P* for each loci can be found in Supplementary File 1.

The 3D scatter plots (of *x* = PC1, *y* = PC2, and *z* = -log_10_*P*) highlighted clusters of significant markers that resembled the peak tops of the QTLs in Manhattan plot distributions, despite the lack of physical positions on a chromosome (Figure 4). It was observed that all clusters included significant markers from all the three trial locations. Two clusters of markers were identified for respectively contents of glucose, xylose, lignin (ADL, KL), and bast fiber content, while only a single cluster of markers was identified for respectively contents of mannose and glucuronic acid.

### QTL Regions Across Locations for Fiber Quality

The correlation analyses across locations highlighted 16 common QTLs across two or three locations, as detailed in Table 3. Eight QTLs were identified across two test locations. Among them, one for respectively contents of ADL (QTL_ADL2_), KL (QTL_KL3_), mannose (QTL_Man2_), and bast fiber content (QTL_BCD2_); and two for respectively contents of glucose (QTL_Glc2_, QTL_Glc3_) and glucuronic acid (QTL_GlcA2_, QTL_GlcA3_). In total, eight QTLs were identified across the three locations, one for respectively contents of ADL (QTL_ADL1_), glucose (QTL_Glc1_), glucuronic acid (QTL_GlcA1_), mannose (QTL_Man1_), xylose (QTL_Xyl1_), and bast fiber content (QTL_BCD1_); and two for content of KL (QTL_KL1_ and QTL_KL2_).

**TABLE 3:** Identification of quantitative trait loci (QTL) regions across locations for seven fiber quality traits of hemp.

QTL Across Locations	Trait	Correlated Representative QTL-Markers Across MultiQTL Models			
		
		CRA	FNPC	VDS	
QTL_ADL1_	ADL%dm	scaffold4782_157368	scaffold80551_6348	scaffold4268_1985	
QTL_ADL2_	ADL%dm	scaffold73277_3647	scaffold34707_3473	–	
QTL_BCD1_	BCD%dm	scaffold56299_8302	scaffold25080_10706	scaffold56299_8302	
QTL_BCD2_	BCD%dm	scaffold13466_34599	–	scaffold3696_33201	
QTL_Glc1_	Glc%dm	scaffold45492_8629	scaffold53823_15026	scaffold18086_2629	
QTL_Glc2_	Glc%dm	scaffold15962_51386	scaffold118257_4014	–	
QTL_Glc3_	Glc%dm	–	scaffold137175_654	scaffold69515_8050	
QTL_GlcA1_	GlcA%dm	scaffold39478_12834	scaffold90847_19970	scaffold55265_18829	scaffold79841_7656
QTL_GlcA2_	GlcA%dm	scaffold111279_1125	–	scaffold28137_3788	
QTL_GlcA3_	GlcA%dm	scaffold1838_41611	scaffold15717_124591	–	
QTL_KL1_	KL%dm	scaffold45492_8629	scaffold69322_5540	scaffold53823_15026	scaffold51533_356
QTL_KL2_	KL%dm	scaffold10943_380	scaffold63415_1601	scaffold13365_3919	
QTL_KL3_	KL%dm	–	scaffold6550_126605	scaffold12000_76415	
QTL_Man1_	Man%dm	scaffold47229_16597	scaffold2731_11599	scaffold34580_21508	scaffold90847_19970
QTL_Man2_	Man%dm	C32103569_1584	–	scaffold32213_3500	
QTL_Xyl1_	Xyl%dm	scaffold30777_901	scaffold42215_3622	scaffold55265_18829	

*QTLs common across locations were identified by correlation analyses (*r* ≥ 0.3, ∼*r*^2^ ≥ 0.1). QTL across locations = name given to the QTL common across locations; Trait: ADL%dm, acid detergent lignin content; BCD, bast fiber content after decortication; Glc%dm, glucose content; GlcA%dm, glucuronic acid content; KL%dm, Klason lignin content; Man%dm, mannose content; and Xyl%dm, xylose content; CRA, representative QTL-markers from MultiQTL models from CRA, Italy; FNPC, representative QTL-markers from MultiQTL models from FNPC, France; and VDS, representative QTL-markers from MultiQTL models from VDS, Netherlands.*

Furthermore, several QTLs across locations for different traits shared some representative QTL-markers. This is the case of QTL_Glc1_ for glucose content and QTL_KL1_ for lignin content that shared the representative QTL-marker scaffold53823_15026. QTL_Xyl1_ for xylose content and QTL_GlcA1_ for glucuronic acid also shared a representative QTL-marker (scaffold55265_18829; Tables 2, 3). The common scaffolds between QTLs for several traits indicated co-localization of some QTLs in tightly close genomic regions.

### Candidate Genes for Fiber Quality of Hemp

In total, 166 transcripts were identified in scaffolds associated to the QTLs and 102 of them showed sequence homology to annotated structural genes from other species (Supplementary Table 5). These genes were involved in several plant physiological processes, including metabolism of proteins, lipids, carbohydrates (monosaccharides and polysaccharides), and lignin. Housekeeping genes, transcription factors and genes involved in nuclear processes (e.g., transport and regulation of chromatin and DNA) were also identified. Moreover, among the annotated transcripts were identified genes involved in plant defense mechanisms against pathogens, genes involved in redox reactions and genes involved in perception of light, such as phytochromes. Finally, transporters of magnesium, potassium and calcium; genes involved in plasmodesmata and genes involved in the transport of vesicles in the cytoplasm were also identified. Among the 102 transcripts, 12 genes showed high similarity to genes involved in cell wall biosynthesis. These candidate genes were identified in six QTLs across locations, as detailed in Table 4.

**TABLE 4:** Putative candidate genes in quantitative trait loci (QTLs) across locations associated to fiber quality traits.

QTL Across Locations	Trait	Scaffold	Candidate Gene (Transcript)	Description	Class of Metabolism	UniProt Code	References
QTL_ADL1_	ADL%dm	scaffold4782	FN14269	*Polygalacturonase*	Polysaccharide	B9SPH9 and A0A1J3JMW1	Chan et al., 2010; Blande et al., 2017
			FN26643	*2-Phytyl-1,4-beta-naphthoquinone methyltransferase*	Lignin	W9RCM0 and A0A1R3I6R5	He et al., 2013
QTL_Glc1_	Glc%dm	scaffold53823	FN16068	*Cytochrome b*_5_	Lignin	A0A1R3IKW6, W9R7N2 and W9R7N2	He et al., 2013
QTL_Glc3_	Glc%dm	scaffold137175	FN29173	*Phosphoenolpyruvate carboxylase (pepc), housekeeping isozyme*	Monosaccharide	W9RZ79	He et al., 2013
QTL_GlcA1_	GlcA%dm	scaffold55265	FN02726	*p-Coumaroyl shikimate 3-hydroxylase (c3h1)*	Lignin	A0A068FPP3	Kamdee et al., 2014
QTL_GlcA3_	GlcA%dm	scaffold15717	FN00563	*Glyceraldehyde-3-phosphate dehydrogenase (gapdh)*	Monosaccharide	W9R8N5	He et al., 2013
			FN19953	*Chalcone synthase (chs)*	Lignin	A0A068PX04, Q1G6S4, Q94LW8 and R9WQY5	Lihova et al., 2006
			FN08380	*Stilbene synthase (sts)*	Lignin	Q6BAR5	Richter et al., 2005
			FN15157	*Alpha-mannosidase*	Polysaccharide	A0A087G6Y0 and A0A1S2XQU0	Parween et al., 2015
QTL_KL1_	KL%dm	scaffold53823	FN16068	*Cytochrome b*_5_	Lignin	A0A1R3IKW6 and W9R7N2	He et al., 2013
QTL_KL2_	KL%dm	scaffold10943	FN05922	*Methyltransferase*	Lignin/Polysaccharide	W9S3D7	He et al., 2013
QTL_KL3_	KL%dm	scaffold6550	FN11795	*Glucan endo-1,3-beta-glucosidase 3/F-box* protein SKIP28	Polysaccharide	W9SPC5, A0A1S2YDV5 and A0A1S3UV78	He et al., 2013; Kang et al., 2014; Parween et al., 2015
QTL_Man1_	Man%dm	scaffold34580	FN16350	*Glucose-1-phosphate adenylyltransferase (glgc)*	Monosaccharide	W9R328	He et al., 2013
QTL_Xyl1_	Xyl%dm	scaffold55265	FN02726	*p-Coumaroyl shikimate 3-hydroxylase (c3h1)*	Lignin	A0A068FPP3	Kamdee et al., 2014

*QTL across locations, name given to the common QTL region across locations; Trait: ADL%dm, acid detergent lignin content; BCD, bast fiber content after decortication; Glc%dm, glucose content; GlcA%dm, glucuronic acid content; KL%dm, Klason lignin content; Man%dm, mannose content; and Xyl%dm, xylose content; Scaffold, genomic scaffold of canSat3, GAC_000230575.1 that composes the QTL across locations (Table 3); Candidate gene, transcript of hemp cultivar ‘Finola’ (transcriptome assembly; Finola) located in a QTL across locations, annotated for a putative function in cell wall metabolism and/or fiber quality (Blast2go; gene description, relevant research papers); Description, description of hemp transcript, as found by Blast2Go annotation, based on homology to sequence from GenBank with known or predicted functions; UniProt code, protein code of the gene. *Arabidopsis* homologues for the candidate genes can be found in Supplementary Table 6.*

A *polygalacturonase* gene, with reported activity in pectin hydrolysis, and *2-phytyl-1,4-*β*-naphthoquinone methyltransferase*, with reported activity in flavonoid biosynthesis, were identified in QTL_ADL1_ for ADL content. Two genes involved in lignin and sugar metabolism were identified in QTLs associated to glucose content: a *cytochrome b5* in QTL_Glc1_ and a *phosphoenolpyruvate carboxylase* (*pepc*) in QTL_Glc3_. In addition, *cytochrome b5* was also identified in QTL_KL1_ for lignin content. This gene was mapped in the scaffold53823 where both QTL_Glc1_ and QTL_KL1_ are located. The lignin-related gene *p-coumaroyl shikimate 3-hydroxylase (c3h1)* was also identified in a scaffold (scaffold55265) covered by QTLs for two different traits. *C3h1* was identified in QTL_GlcA1_ for glucuronic acid content and in QTL_Xyl1_ for xylose content. Two genes involved in sugar metabolism (*glyceraldehyde-3-phosphate dehydrogenase* (*gapdh*) and α*-mannosidase*) and two genes indirectly involved in the lignin metabolism, [*chalcone synthase* (*chs*) and *stilbene synthase* (*sts*)], primarily involved in the precursor pathways of flavonoid and stilbene biosynthesis, were identified in QTL_GlcA3_ for glucuronic acid content. A *methyltransferase* gene involved in the biosynthesis of many cell wall components was identified in QTL_KL2_ and a *glucan endo-1,3-beta-glucosidase 3* gene was identified in QTL_KL3_. Finally, a *glucose-1-phosphate adenylyltransferase* (*glgc*) involved in the metabolism of monosaccharides was identified in QTL_Man1_ for mannose content.

## Discussion

### Genetic Architecture Underlying Hemp Fiber Quality

The study of the genetic architecture of hemp fiber quality is fundamental to develop molecular strategies to breed for new hemp cultivars with improved fiber properties. The genetics of hemp fiber quality have been poorly studied and genetic markers for these traits are lacking in hemp. Nonetheless, GWAS analyses have proved to be a powerful approach to detect genetic components controlling quantitative traits. GWAS analyses were used to detect QTLs for agronomic traits, biomass content and cell wall composition in several fiber crops, such as cotton (Liu et al., 2018) and flax (Xie et al., 2018).

In the present study, we developed a modified GWAS approach to identify and map QTLs in hemp. In the absence of a complete genome sequence it is difficult to map the boundaries of QTLs and to identify independent QTLs (Sawler et al., 2015). To overcome this limitation, we developed a MultiQTL approach based on the levels of explained variance and the correlations between significant markers. This approach was applied in hemp and several QTLs for fiber quality traits were successfully identified. This method enables genome-wide association analyses for hemp, and it can be extended to genetic studies in other orphan species, for which no complete and assembled genome is available (Graham, 2013).

All fiber quality traits assessed in this study were largely heritable across different environments (*H*^2^ = 0.88–0.96) and the rankings of the accessions were similar at the three locations for all traits (Supplementary Table 4; Petit et al., 2020). Only glucuronic acid was largely influenced by the environment (Supplementary Table 4). Petit et al. (2020) suggested that the content of this monosaccharide might have stronger general response to adapt to the environment, independently of the heritable genetic control of the trait. This might be explained by functional redundancy between different cell wall components. The putative changes in plant fitness associated to the changes in glucuronic acid content across environments might be replaced by other cell wall components with similar functions, such as lignin (Sarkar et al., 2009). Despite the large environmental component of glucuronic acid, as it phenotypic variation is also explained by the genetic factors, it is also a good candidate for genetic association studies. Genetic studies on cell wall composition in other crops also showed large heritabilities for biomass quality related traits. In maize, heritabilities for biomass traits, such as contents of cellulose, hemicellulose and lignin were reported above 0.6 (Torres et al., 2015). In miscanthus, the heritabilities of these traits ranged from 0.4 to 0.72 (Slavov et al., 2014; van der Weijde et al., 2017). These high heritabilities indicate that the genetic gains of fiber quality can be maximized in several crops.

In this report, sixteen QTLs across locations were found to be associated to fiber quality in hemp. For six of these QTLs, cell wall candidate genes were identified. Among those were genes involved in the metabolism of monosaccharides, genes involved in the metabolism of polysaccharides, genes involved in the metabolism of glycoproteins and genes involved in lignin biosynthesis. The genes *phosphoenolpyruvate carboxylase* (*pepc*), *glyceraldehyde-3-phosphate dehydrogenase* (*gapdh*) and g*lucose-1-phosphate adenylyltransferase (glgc*), involved in sugar metabolism were identified underneath QTL_Glc3_, QTL_GlcA3_, and QTL_Man1_, respectively. *Phosphoenolpyruvate carboxylase* (*pepc*) codifies for an enzyme that catalyzes the carboxylation of phosphoenolpyruvate to oxaloacetate to produce malate. The activity of this enzyme has been reported to play multiple roles, among them fiber elongation, carbon storage and energy production (Zieher, 2010). The accumulation of malate and sugars, is thought to play an important role in the fiber elongation, through osmotic regulation and charge balance. For instance, Li et al. (2010) reported in cotton that in periods of rapid elongation phase, *pepc* genes were highly expressed, but weakly expressed at slow elongation periods. In addition, *pepc* is involved in carbon storage and energy production by converting phosphoenolpyruvate into oxaloacetate (Jeanneau et al., 2002). Phosphoenolpyruvate results from the glycolysis of glucose and consequently reduces the source of monosaccharides for other pathways, such as to biosynthetise cell wall polysaccharides. *Glyceraldehyde-3-phosphate dehydrogenase* (*gapdh*) is another important gene involved in glycolysis. This enzyme catalyzes the conversion of glyceraldehyde-3-phosphate to 1,3-bisphosphoglycerate with reduction of NAD^+^ to NADH. Downregulation of *gapdh* in *Arabidopsis* resulted in drastic changes in the sugar balance of the plant, arrested root development and dwarfism (Muñoz-Bertomeu et al., 2009). This study showed the importance of *gapdh* in plant primary metabolism. Thus, variation in *gapdh* expression might change the monosaccharide availability for different cellular biosynthetic processes. G*lucose-1-phosphate adenylyltransferase (glgc*) is involved in the partitioning of α-D-glucose-1P for starch and cell wall polysaccharide biosynthesis. *Glucose-1-phosphate adenylyltransferase* codes for an enzyme that catalizes the conversion of α-D-glucose-1P to ADP-glucose. ADP-glucose will be further used for starch biosynthesis (de Setta et al., 2014). Therefore, this enzyme competes for sustrate with enzymes involved in the conversion of α-D-glucose-1P to UDP-glucose (Lozovaya et al., 1996), that will be further used for the biosynthesis of cell wall polysaccharides. UDP-glucose serves as a direct source of UDP-galactose, UDP-rhamnose, and UDP-glucuronic acid; and an indirect source of UDP-xylose, UDP-galacturonic acid, and UDP-apiose (Wierzbicki et al., 2019).

*Alpha-mannosidase*, g*lucan endo-1,3-*β*-glucosidase 3*, and *polygalacturonase* are involved in the metabolism of glycoproteins and polysaccharides. *Alpha-mannosidase* codes for an enzyme involved in early N-glycan processing. The N-glycans are oligosaccharides (Glc_3_Man_9_GlcNAc_2_) linked to nitrogen (N) and involved in the biosynthesis of N-glycoprotein (Liebminger et al., 2009). Cell wall N-glycoproteins are important structural components of the cell walls (Roberts et al., 1985; Nguema-Ona et al., 2014). They are a little understood group of proteins that play a diversity of functions, including signaling and interacting with the surrounding environment; and plant defense. Moreover, they are essential for plant development and responses to stress (Nguema-Ona et al., 2014; Strasser, 2014). In *Arabidopsis*, three α*-mannosidase* genes have been identified, named as *mns* genes. *Arabidopsis* mutants for *mns* genes resulted in the formation of aberrant N-glycan. N-glycoproteins are incorrectly folded, affecting the function of the proteins (Nguema-Ona et al., 2014). The mutant plants displayed short, swollen roots and altered cell walls. *Arabidopsis* mutant for one, two or three *mns* genes displayed lower amount of homogalacturonan-type pectin. Consequently, Nguema-Ona et al. (2014) suggested that N-glycosylation of glycoproteins plays a role in the correct targeting, assembly, or stability of cell wall biosynthetic or remodeling enzymes. Furthermore, the cell wall is a highly dynamic structure with constant synthesis and degradation of cell wall components throughout plant development (Houston et al., 2016). Degradation of cell wall components is an important step during pathogenesis. It is known that pathogens trigger the expression of endogenous plant genes that induce a degradation of the cell wall. Therefore, genes involved in cell wall associated defense can modify cell wall composition [reviewed in Underwood (2012)]. *Glucan endo-1,3-*β*-glucosidase 3* codes for an enzyme involved in degradation of cell wall polysaccharides and plant defense against pathogens. In *Arabidopsis*, this enzyme has been implicated in pathogenesis when the plants were infected with tobamovirus (Zavaliev et al., 2013). The infection triggered the endogenous secretion of this enzyme in the cell wall, which degraded callose in plasmodesmata. Consequently, the damage in the cell wall enhanced the virus spread. *Arabidopsis* mutants for this gene retained the enzyme in the endoplasmic reticulum and was not secreted. Zavaliev et al. (2013) observed that the cell walls of the mutant plants were unaffected. As a result, alterations in expression or function of *glucan endo-1,3-*β*-glucosidase 3* gene is likely to affect the dynamism and architecture of the cell walls. *Polygalacturonases* are another group of genes coding for enzymes involved in degradation of the cell wall, specifically homogalacturonan type of pectin. These enzymes function in a wide range of developmental processes, including cell separation and cell elongation to control the shape of the plant architecture (Gonzalez-Carranza et al., 2007; Babu and Bayer, 2014). They play important roles in the primary cell wall of meristematic and elongating cells, before secondary cell walls can fortify the fibers (Zhong and Ye, 2007). In *Arabidopsis*, 69 *polygalacturonases* has been identified and the alteration of several of those was shown to produce aberrations in plant developmental processes [reviewed in Babu and Bayer (2014)]. For instance, *polygalacturonase involved in expansion1 (pgx1)* was shown to be involved in hypocotyl elongation. *Arabidopsis pgx1* mutants showed strong cell-elongation defects, alterations in pectin molecular mass and cell wall composition (Xiao et al., 2014).

*Cytochrome b*_5_, *p-coumaroyl shikimate 3-hydroxylase* (*c3h1*), *chalcone synthase* (*chs*), *stilbene synthase* (*sts*), and *2-phytyl-1,4-*β*-naphthoquinone methyltransferase* are involved directly or indirectly in lignin biosynthesis. A recent study in *Arabidopsis* described the product of *cytochrome b*_5_ as an obligate electron shuttle protein specific for the biosynthesis of syringyl lignin subunit (Gou et al., 2019). The study revealed that the *Arabidopsis* mutant for *cytochrome b*_5_ suppressed the catalytic activity of the enzymes cinnamic acid 4-hydroxylase (C4H) and ferulate 5-hydroxylase (F5H). These two enzymes are essential in the biosynthesis of lignin and the suppression of their activity affects lignin content (Boerjan et al., 2003; Sykes et al., 2015). *P-coumaroyl shikimate 3-hydroxylase* is another essential gene in the lignin biosynthetic pathway (Boerjan et al., 2003). In eucalyptus and alfalfa, the down-regulation of c*3h1* expression displayed lower lignin content (Ralph et al., 2006, 2012). In addition, in poplar, the repression of c*3h1* was associated to alterations in the cell wall and in improved sugar release (Sykes et al., 2015). Moreover, these studies showed that the repression of c*3h1* did not lead to deleterious impacts on plant growth. These results indicate positive implications for increasing fiber quality without affecting plant fitness. Furthermore, anthocyanin biosynthesis and monolignol production share the phenylpropanoid pathway from phenylalanine to *p*-coumaroyl CoA. The latter is directly used in the flavonoid pathway through *chalcone synthase*, or used in the monolignol pathway through genes directly involved in the lignin metabolism (Behr et al., 2016). *Stilbene synthase* is another gene involved in the flavonoid and stilbene biosynthesis. Overexpression of genes involved in the production of flavonoids might decrease the availability of metabolites for the monolignol pathway, which seems to be associated to differences in lignification of hemp fibers. These genes are known to be regulated at the level of transcription. MYB transcription factors (e.g., AtMYB75) are key regulators of the anthocyanin branch of the phenylpropanoid pathway to favor flavonoid biosynthesis at the expense of monolignol biosynthesis [Zhong and Ye, 2009 and Bhargava et al., 2010 (*Arabidopsis*); Shaipulah et al., 2016 (petunia)]. External treatment of chalcone in soybean was reported to inhibit lignin biosynthesis (Chen et al., 2011). In addition, Behr et al. (2016) reported differential expression of *chalcone synthase* gene between young and old hypocotyls of hemp. *Chalcone synthase* gene was more expressed in young hypocotyls to produce anthocyanins, acting as a photo-protectant when the hypocotyl emerges from the soil (Steyn et al., 2002; Wang et al., 2012). In older hypocotyls the expression of *chalcone synthase* gene is reduced to allow the biosynthesis of monolignol from *p*-coumaroyl CoA. This suggests that variation in key genes of chalcone (*chs*) and stilbene (*sts*) biosynthesis are responsible for variation in lignin content (Vannozzi et al., 2012). *2-phytyl-1,4-*β*-naphthoquinone methyltransferase* is an enzyme involved in the biosynthesis of phylloquinone. This gene plays an active role in the biosynthesis of aromatic amino acids, such as phenylalanine (reviewed in Tzin and Galili, 2010). Variation in *2-phytyl-1,4-*β*-naphthoquinone methyltransferase* might affect the lignin content similarly to *chalcone* and *stilbene genes*. They might affect the biosynthesis of monolignols and consequently the lignification of the fibers. These results are supported by the revisions of Boerjan et al. (2003) that reported profound effects on the biosynthesis of lignin due to availability of phenylalanine.

Moreover, the identification of the same genomic scaffolds in QTLs for different traits is an indication of co-localization of QTLs for fiber quality in tightly close genomic regions. For instance, QTL_Glc1_ and QTL_KL1_ for respectively glucose and lignin content shared a genomic scaffold. Co-localization of scaffolds was also found in QTL_Xyl1_ and QTL_GlcA1_ for respectively xylose and glucuronic acid content (Table 3). Cell wall composition is a complex trait and many cell wall components are highly interconnected. Petit et al. (2019) reported large correlations between cell wall components in hemp, such as a negative correlation between the contents of glucose and lignin (*r* = −0.93, which reflects the relationship between bast and woody hemp core, as glucose is the main component of bast and lignin of woody hemp core) and contents of xylose and glucuronic acid (*r*^2^ = 0.96; Petit et al., 2020). The large correlation between different cell wall components is an indication that QTLs affecting a trait, will automatically affect other correlated cell wall traits. The similar phenotypic variation between correlated traits is thus associated in the same genomic regions. For instance, the gene *cytochrome b*_5_, identified in the genomic scaffold common for glucose and lignin content, can directly affect lignin content. The alteration in lignin content can then indirectly affect the content of glucose. These results might be explained in the context of hemp stem development. The identified *cytochrome b*_5_ might be a reflection of the differential gene expression between young and old hypocotyls of hemp (Behr et al., 2016). Behr et al. (2016) identified larger number of genes related to lignin metabolism in young than in old hypocotyls of hemp. In addition, Novaes et al. (2010) reviewed evidences that the negative correlation between lignin and cellulose (glucose) is partially mediated at the level of transcription. The overproduction of a cell wall component can inhibit the production of another component, through transcription factors mediating different pathways [e.g., NYB46, MYB83, and secondary wall NAC (SWN)] (Zhong and Ye, 2012). Another example of co-localization is the QTLs for contents of xylose and glucuronic acid. These monosaccharides are main components of xylans in eudicots plants (Pauly et al., 2013). Therefore, the variation of one component affects the variation of the other one. Moreover, *c3h1* gene for lignin biosynthesis was identified in the common QTL between xylose and glucuronic acid. Lignin and xylans are cross-linked (Pauly et al., 2013) and highly correlated (Petit et al., 2020). Allelic variation of *c3h1* can directly lead to phenotypic variation of lignin content, as reported in other crops (Ralph et al., 2006, 2012; Sykes et al., 2015). Thereafter, alteration of lignin content can affect the cross-linking to other polymers and indirectly the contents of xylose and glucuronic acid. An evidence for this are the studies that report modifications of xylans (e.g., methylations of glucuronic acid) affecting lignin composition [reviewed in Wierzbicki et al. (2019)].

The candidate genes identified in this study are of great significance for further studies, including reverse genetic approaches, such as candidate gene functional studies, but specially differential expression analysis from different stem tissues. Such studies would be very valuable for understanding the molecular mechanisms responsible for hemp fiber quality.

### Importance of QTLs in Genetic Improvement of Hemp

The identification of QTLs across locations for hemp fiber quality will have relevant implications for the breeding programs of the crop. Breeding for QTLs identified across two locations will lead to genotypes that perform well under certain environments but not necessarily under other environments. Furthermore, when breeding for QTLs identified across the three locations, the advantage is that the resulting improved genotypes will perform well under more environments. The two or three markers for each QTL across locations would be the first candidates to include in marker assisted selection (MAS) breeding schemes in hemp. The implementation of molecular markers in hemp breeding programs is expected to speed up the development of new hemp cultivars with improved fiber properties. The use of molecular markers has several advantages, including the early stage selection of promising plants. In addition, the use of markers circumvents the phenotyping of large number of samples for fiber quality, which usually involves large costs and time in breeding programs.

Furthermore, the identification of QTLs and candidate genes for fiber quality, using the hemp panel, indicates that this panel was large enough to include an extensive range of genotypic and phenotypic variation for genetic studies in hemp. The large variability of the hemp panel enables further mapping studies for hemp traits that still remain poorly studied. The hemp panel and the methodology developed in this study are a great value to extend the genetic basis of many traits beyond cell wall traits, such as flowering time and sex determination (Salentijn et al., 2019) and understand their interaction with fiber quality traits. Those studies would also provide more molecular breeding tools to accelerate the development of new hemp cultivars with improved fiber quality.

## Conclusion

The present study provides valuable insights into the genetic and molecular architecture of fiber quality in hemp, which advocates for positive prospects to modernize breeding programs of hemp toward molecular approaches. The sixteen QTLs for fiber quality are the first candidates to include in marker assisted selection breeding schemes of hemp. The implementation of these QTLs in molecular breeding programs in hemp will accelerate the selection of interesting individuals and will bypass the phenotyping of large number of samples, leading to important cost reductions. In addition, the identification of QTLs and candidate genes for fiber quality enables the use of the hemp panel in further studies to extend the genetic architecture of other important traits in hemp. Furthermore, the correlation method used in this study to identify non-collinear QTLs can be extended to genetic studies in other orphan species, for which no complete genome sequence is available.

## Data Availability Statement

RADseq data, selected informative SNP markers and the annotated transcriptomic data of the hemp cultivar *C. sativa* ‘Finola’ are deposited in the 4TU.ResearchData archive (https://doi.org/10.4121/12826832, https://doi.org/10.4121/uuid:d17c382c-f292-4875-888b-47e6433d600f).

## Author Contributions

JP designed and performed the experiments, analyzed the data, and wrote the manuscript. M-JP and EL helped analyzing the data and revised the manuscript. ES helped designing and performing the experiments, helped analyzing the data, and revised the manuscript. CD helped designing and performing the experiments and revised the manuscript. LT coordinated and supervised this study, experimental strategy, and discussion of the outcomes and revised the manuscript. All authors contributed to the article and approved the submitted version.

## Conflict of Interest

The authors declare that the research was conducted in the absence of any commercial or financial relationships that could be construed as a potential conflict of interest.

## Footnotes

1 http://www.r-project.org/

2 https://doi.org/10.4121/uuid:d17c382c-f292-4875-888b-47e6433d600f
