# Supplementary material for: Elucidating the Genetic Architecture of Fiber Quality in Hemp (Cannabis sativa L.) Using a Genome-Wide Association Study
Source: Front Genet. 2020 Sep 17;11:566314. doi: 10.3389/fgene.2020.566314 (PMC7527631; doi:10.3389/fgene.2020.566314)
Supplement: Supplementary file 1 [file Data_Sheet_1.docx]

**Supplementary Table 1.** Panel of 123 hemp (*Cannabis sativa* L.) accessions from Petit et al. (2020). All accessions were cultivated in three locations across Europe as specified in Petit et al. (2020). Population types B, L and W stand for breeding material, landraces and wild material, respectively. Accession type are based on use.

| MultiHemp code | Accession name / Code | Origin | Accession type | Population type | Provider |
| --- | --- | --- | --- | --- | --- |
| MH-AGM-701 | Fibrol / - | Hungary | Fibre | B | AGM |
| MH-AGM-702 | Tiborszallasi / - | Hungary | Fibre | B | AGM |
| MH-AGM-703 | Tisza / - | Hungary | Fibre | B | AGM |
| MH-AGM-704 | KC Dora / - | Hungary | Fibre | B | AGM |
| MH-AGM-705 | Monoica / - | Hungary | Fibre | B | AGM |
| MH-CAAS-601 | CYM171 / - | China | Fibre | B | CAAS |
| MH-CAAS-602 | CYM28 / - | China | Fibre | B | CAAS |
| MH-CAAS-603 | Yunma 5 / - | China | Fibre | B | CAAS |
| MH-CAAS-604 | CYM49 / - | China | Fibre | B | CAAS |
| MH-CAAS-605 | CYM273 / - | China | Fibre | B | CAAS |
| MH-CRA-401 | CRA_1 / - | Italy | Fibre | B | CRA |
| MH-CRA-402 | CRA_2 / - | Italy | Fibre | B | CRA |
| MH-CRA-404 | Delta llosa / - | Spain | Fibre | B | CRA |
| MH-CRA-405 | CRA_4 / - | Italy | Fibre | B | CRA |
| MH-CRA-406 | Carma Monoica / - | Italy | Fibre | B | CRA |
| MH-CRA-407 | Supermono / - | Italy | Fibre | B | CRA |
| MH-CRA-408 | Fibranova (CRA_5) / - | Italy | Fibre | B | CRA |
| MH-CRA-409 | Carmagnola / - | Italy | Fibre | B | CRA |
| MH-CRA-410 | Ermes A / - | Italy | Fibre | B | CRA |
| MH-CRA-411 | CS (CRA_6) / - | Italy | Fibre | B | CRA |
| MH-CRA-412 | Carmaleonte / - | Italy | Fibre | B | CRA |
| MH-CRA-413 | CRA_7 / - | Italy | Fibre | B | CRA |
| MH-CRA-414 | W-1 / - | Italy | Fibre | B | CRA |
| MH-CRA-415 | Zenit / - | Romania | Fibre | B | CRA |
| MH-CRA-416 | Denise / - | Romania | Fibre | B | CRA |
| MH-CRA-417 | CRA_8 / - | Italy | Fibre | B | CRA |
| MH-CRA-418 | SVGB-10611 / - | Italy | Fibre | B | CRA |
| MH-CRA-419 | USO 14 Monoica / - | Ukraine | Fibre | B | CRA |
| MH-CRA-420 | USO 31 / - | Ukraine | Fibre | B | CRA |
| MH-FNPC-201 | - / A11-121-1 | France | Fibre | B | FNPC |
| MH-FNPC-202 | - / A11-121-2 | France | Fibre | B | FNPC |
| MH-FNPC-203 | - / A11-121-3 | France | Fibre | B | FNPC |
| MH-FNPC-204 | - / A11-121-4 | France | Fibre | B | FNPC |
| MH-FNPC-205 | - / A11-121-5 | France | Fibre | B | FNPC |
| MH-FNPC-206 | - / A11-121-6 | France | Fibre | B | FNPC |
| MH-FNPC-207 | - / A11-121-7 | France | Fibre | B | FNPC |
| MH-FNPC-209 | - / A11-121-9 | France | Fibre | B | FNPC |
| MH-FNPC-210 | - / A11-121-10 | France | Fibre | B | FNPC |
| MH-FNPC-211 | - / A11-121-11 | France | Fibre | B | FNPC |
| MH-FNPC-212 | - / A11-121-12 | France | Fibre | B | FNPC |
| MH-FNPC-213 | - / A11-121-13 | France | Fibre | B | FNPC |
| MH-FNPC-214 | - / A11-121-14 | France | Fibre | B | FNPC |
| MH-FNPC-215 | - / A11-121-15 | France | Fibre | B | FNPC |
| MH-FNPC-216 | - / A11-121-16 | France | Fibre | B | FNPC |
| MH-FNPC-217 | - / A11-121-17 | France | Fibre | B | FNPC |
| MH-FNPC-218 | - / A11-121-18 | France | Fibre | B | FNPC |
| MH-FNPC-219 | - / A11-121-19 | France | Fibre | B | FNPC |
| MH-FNPC-220 | - / A11-121-20 | France | Fibre | B | FNPC |
| MH-FNPC-221 | - / A11-121-21 | France | Fibre | B | FNPC |
| MH-FNPC-222 | - / A11-121-22 | France | Fibre | B | FNPC |
| MH-FNPC-223 | - / A11-121-23 | France | Fibre | B | FNPC |
| MH-FNPC-224 | - / A11-121-24 | France | Fibre | B | FNPC |
| MH-FNPC-225 | - / A10-122-1 | France | Fibre | B | FNPC |
| MH-FNPC-226 | - / A10-122-2 | France | Fibre | B | FNPC |
| MH-FNPC-227 | - / A10-122-4 | France | Fibre | B | FNPC |
| MH-FNPC-228 | - / A103-122-1 | France | Fibre | B | FNPC |
| MH-FNPC-229 | - / A103-122-2 | France | Fibre | B | FNPC |
| MH-FNPC-230 | - / A103-122-3 | France | Fibre | B | FNPC |
| MH-FNPC-231 | - / A103-122-4 | France | Fibre | B | FNPC |
| MH-FNPC-232 | - / A103-122-6 | France | Fibre | B | FNPC |
| MH-FNPC-233 | - / A103-122-8 | France | Fibre | B | FNPC |
| MH-FNPC-234 | - / A103-122-10 | France | Fibre | B | FNPC |
| MH-FNPC-235 | - / A9-122-1 | France | Fibre | B | FNPC |
| MH-FNPC-236 | - / A9-122-2 | France | Fibre | B | FNPC |
| MH-FNPC-237 | - / A9-122-3 | France | Fibre | B | FNPC |
| MH-FNPC-238 | - / A9-122-4 | France | Fibre | B | FNPC |
| MH-FNPC-239 | - / A102-122-1 | France | Fibre | B | FNPC |
| MH-FNPC-240 | - / A102-122-2 | France | Fibre | B | FNPC |
| MH-FNPC-241 | - / A102-122-3 | France | Fibre | B | FNPC |
| MH-FNPC-242 | - / A102-122-4 | France | Fibre | B | FNPC |
| MH-FNPC-243 | - / A102-111-1 | France | Fibre | B | FNPC |
| MH-FNPC-244 | - / A102-111-2 | France | Fibre | B | FNPC |
| MH-FNPC-245 | - / A7-104-1 | France | Fibre | B | FNPC |
| MH-FNPC-246 | - / A7-105-4 | France | Fibre | B | FNPC |
| MH-FNPC-248 | - / B6-093-3 | France | Fibre | B | FNPC |
| MH-FNPC-250 | - / B6-093-17 | France | Fibre | B | FNPC |
| MH-FNPC-251 | Férimon / - | France | Fibre | B | FNPC |
| MH-FNPC-252 | Fédora 17 / - | France | Fibre | B | FNPC |
| MH-FNPC-253 | Félina 32 / - | France | Fibre | B | FNPC |
| MH-FNPC-254 | Epsilon 68 / - | France | Fibre | B | FNPC |
| MH-FNPC-255 | Futura 75 / - | France | Fibre | B | FNPC |
| MH-FNPC-256 | Santhica 27 / - | France | Fibre | B | FNPC |
| MH-IWNRZ-901 | Bialobrzeskie / - | Poland | Fibre | B | IWNRZ |
| MH-IWNRZ-902 | Beniko / - | Poland | Fibre | B | IWNRZ |
| MH-IWNRZ-903 | Tygra / - | Poland | Fibre | B | IWNRZ |
| MH-LARC-501 | Katlakalna / - | Latvia | Fibre | B | LARC |
| MH-UOY-801 | Finola / - | Finland | Seed | B | UOY |
| MH-VDS-301 | Chameleon / - | Netherlands | Fibre | B | VDS |
| MH-VDS-302 | Marcello / - | Netherlands | Fibre | B | VDS |
| MH-VDS-303 | Markant / - | Netherlands | Fibre | B | VDS |
| MH-VDS-304 | Ivory / - | Netherlands | Fibre | B | VDS |
| MH-WU-101 | JSO 16 / 891229 | Russia | Fibre | B | WUR |
| MH-WU-102 | Ajkai-A-TF / 891054 | Hungary | Fibre | L | WUR |
| MH-WU-103 | Fibrimon 56 / 880828 | France | Fibre | B | WUR |
| MH-WU-104 | Rastislavicke / 880816 | Slovakia | Fibre | B | WUR |
| MH-WU-105 | Krasnodarskaja 56 / 891333 | Ukraine | Fibre | B | WUR |
| MH-WU-106 | Dneprovskaja 84 / 921054 | Russia | Fibre | L | WUR |
| MH-WU-107 | - / 883290 | Russia | Fibre | L | WUR |
| MH-WU-108 | Lovrin 110 / 883173 | Romania | Fibre | B | WUR |
| MH-WU-109 | Bialobrzeskie / 891223 | Poland | Fibre | B | WUR |
| MH-WU-110 | - / 880973 | Spain | - | - | WUR |
| MH-WU-111 | Kompolti Sargászáru / 883049 | Hungary | Fibre | B | WUR |
| MH-WU-112 | - / 883262 | Spain | - | - | WUR |
| MH-WU-113 | Kompolti hybrid TC / 891070 | Hungary | Fibre | B | WUR |
| MH-WU-114 | Fibrimon 56 / 891158 | France | Fibre | B | WUR |
| MH-WU-115 | - / 921203 | Canada | - | W | WUR |
| MH-WU-116 | Panorama var. globosa / 910914 | Hungary | Ornamental | B | WUR |
| MH-WU-117 | Silistrenski / 901107 | Bulgaria | Fibre | B | WUR |
| MH-WU-118 | Csehslovák-A-TF / 891068 | Slovakia | Fibre | - | WUR |
| MH-WU-119 | - / 891288 | Poland | Fibre | - | WUR |
| MH-WU-120 | - / 891090 | Turkey | - | L | WUR |
| MH-WU-121 | Komoroi-A-TF / 891046 | Hungary | Fibre | L | WUR |
| MH-WU-122 | - / 883289 | Russia | Fibre | L | WUR |
| MH-WU-123 | Juznaja Odnovremenno / 883293 | Russia | Fibre | B | WUR |
| MH-WU-124 | - / 891240 | Spain | - | - | WUR |
| MH-WU-125 | Orosi-A-TF / 891059 | Hungary | Fibre | - | WUR |
| MH-WU-126 | Kompolti / 883048 | Hungary | Fibre | - | WUR |
| MH-WU-127 | Dneprovskaja odnodomnaja 6 / 891326 | Ukraine | Fibre | B | WUR |
| MH-WU-128 | - / 891327 | - | Fibre | L | WUR |
| MH-WU-129 | Superfibra / 883040 | Italy | Fibre | B | WUR |
| MH-WU-130 | - / 891057 | Hungary | - | L | WUR |
| MH-WU-131 | - / 891094 | Turkey | - | L | WUR |
| MH-WU-132 | - / 880817 | Germany | - | - | WUR |

**Supplementary Table 2.** Number of QTLs and explained variances of the multiQTL models for the content of glucose in the three locations under different putative conditions of independence (r).

| Trait | Location | Correlation (r) | Number of QTLs | Explained variance |
| --- | --- | --- | --- | --- |
| Glucose | CRA | 0.1 | 2 | 30.93 |
| Glucose | CRA | 0.2 | 2 | 29.18 |
| Glucose | CRA | 0.3 | 3 | 29.22 |
| Glucose | CRA | 0.4 | 2 | 29.36 |
| Glucose | CRA | 0.5 | 6 | 47.67 |
| Glucose | CRA | 0.6 | 5 | 65.27 |
| Glucose | CRA | 0.7 | 19 | 84.22 |
| Glucose | FNPC | 0.1 | 2 | 37.42 |
| Glucose | FNPC | 0.2 | 2 | 39.07 |
| Glucose | FNPC | 0.3 | 5 | 64.63 |
| Glucose | FNPC | 0.4 | 5 | 68.22 |
| Glucose | FNPC | 0.5 | 14 | 80.07 |
| Glucose | FNPC | 0.6 | 13 | 84.38 |
| Glucose | FNPC | 0.7 | 6 | 74.78 |
| Glucose | VDS | 0.1 | 3 | 53.23 |
| Glucose | VDS | 0.2 | 3 | 56.17 |
| Glucose | VDS | 0.3 | 3 | 55.72 |
| Glucose | VDS | 0.4 | 6 | 79.21 |
| Glucose | VDS | 0.5 | 6 | 66.4 |
| Glucose | VDS | 0.6 | 14 | 79.26 |
| Glucose | VDS | 0.7 | 8 | 75.62 |

**Supplementary Table 3.** Genetic diversity in seven fibre quality traits of hemp grown in three contrasting locations. Means, standard deviations and coefficient of variation (CV%). Data adapted from (Petit et al., 2019b).

| Trait | Location | | | | | |
| --- | --- | --- | --- | --- | --- | --- |
|  | CRA | | FNPC | | VDS | |
|  | Mean ± SD | CV% | Mean ± SD | CV% | Mean ± SD | CV% |
| Glucose | 49.28 ± 2.63 | 5.33 | 47.74 ± 2.28 | 4.77 | 49.63 ± 2.87 | 5.78 |
| Mannose | 2.93 ± 0.35 | 11.83 | 2.74 ± 0.3 | 10.99 | 2.57 ± 0.37 | 14.40 |
| Xylose | 13.69 ± 1.38 | 10.04 | 12.74 ± 1.16 | 9.10 | 14.33 ± 1.62 | 11.32 |
| Glucuronic acid | 0.31 ± 0.06 | 18.11 | 0.35 ± 0.06 | 15.68 | 0.43 ± 0.07 | 15.48 |
| ADL | 8.7 ± 0.91 | 10.43 | 8.85 ± 0.84 | 9.46 | 9.63 ± 0.97 | 10.05 |
| KL | 14.17 ± 1.21 | 8.51 | 14.14 ± 1.02 | 7.23 | 15.71 ± 1.44 | 9.19 |
| BCD | 31.63 ± 6.4 | 20.23 | 29.6 ± 5.04 | 17.02 | 26.19 ± 6.89 | 26.30 |

CV% = $\frac{Standard deviation}{Mean}*100$.

**Supplementary Table 4.** Variance components and broad-sense heritability (H^2^) of seven fibre quality traits. The variances explained by each component are shown as the proportion of total variance (%). Data adapted from (Petit et al., 2019b).

| Trait | Abbreviation | Trait group | Cell wall | Location (*L*%) | Block within Location (*B*%) | Genotype (*G*%) | Genotype x Enviroment interaction (*GxE*%) | Error (*ε*%) | H^2^ |
| --- | --- | --- | --- | --- | --- | --- | --- | --- | --- |
| Acid Detergent Lignin (%) | ADL%dm | Cell wall | Lignin | 22.52 | 0.98 | 44.14 | 8.72 | 23.64 | 0.89 |
| Glucose (%) | Glc%dm | Cell wall | Cellulose | 11.14 | 1.52 | 67.57 | 4.3 | 15.48 | 0.96 |
| Glucuronic acid (%) | GlcA%dm | Cell wall | Xylan | 46.64 | 1.96 | 29.77 | 2.97 | 18.66 | 0.91 |
| Klasson Lignin (%) | KL%dm | Cell wall | Lignin | 34.04 | 0.45 | 47.73 | 3.31 | 14.47 | 0.95 |
| Mannose (%) | Man%dm | Cell wall | Mannan | 21.24 | 0.53 | 41.62 | 8.24 | 28.36 | 0.88 |
| Xylose (%) | Xyl%dm | Cell wall | Xylan | 23.77 | 0.29 | 54.79 | 4.86 | 16.29 | 0.94 |
| Bast content after decortication (%) | BCD% | Fibre | - | 16.66 | 0.89 | 66.79 | 5.36 | 10.29 | 0.96 |

**Supplementary Table 5.** Transcripts of hemp cultivar ‘Finola’ (van Bakel et al., 2011) and homologous genes detected in the scaffolds composing QTL regions associated to fibre quality in hemp.

| QTL region | Trait | Scaffold | Transcript | Homologous gene | Species | UniProt code |
| --- | --- | --- | --- | --- | --- | --- |
| QTL*_ADL1_* | ADL | scaffold4782 | FN14269 | *Polygalacturonase* | Ricinus communis / Noccaea caerulescens | B9SPH9 / A0A1J3JMW1 |
|  | ADL | scaffold4782 | FN24817 | *Retrovirus-related Pol poly from transposon TNT 1-94* | Cajanus cajan | A0A151RET9 |
|  | ADL | scaffold4782 | FN16188 | *Kinase* | Ricinus communis | B9SPH8 |
|  | ADL | scaffold4782 | FN07726 | *Cysteine-rich receptor kinase 3* | Cephalotus follicularis / Morus notabilis | A0A1Q3B1W2 / W9RKP8 |
|  | ADL | scaffold4782 | FN29181 |  |  |  |
|  | ADL | scaffold4782 | FN20710 | *Cysteine-rich receptor kinase 2* | Nicotiana tabacum | A0A1S4CY69 |
|  | ADL | scaffold4782 | FN26643 | *2-phytyl-1,4-beta-naphthoquinone methyltransferase* | Morus notabilis / Corchorus capsularis / Populus trichocarpa | W9RCM0 / U5G1A9 / A0A1R3I6R5 |
|  | ADL | scaffold4782 | FN07002 | *NSP-interacting kinase 3* | Morus notabilis / Theobroma cacao | W9RKQ2 / A0A061E6D7 |
|  | ADL | scaffold4782 | FN11699 | *Trichome birefringence-like 2* | Capsicum annuum | A0A1U8EN65 |
|  | ADL | scaffold4782 | FN00029 | *Homeobox knotted-1-like 6* | Morus notabilis | W9RCT7 |
|  | ADL | scaffold4782 | FN24332 |  |  |  |
|  | ADL | scaffold80551 | FN12389 | *Importin subunit beta-4* | Morus notabilis | W9RM13 |
|  | ADL | scaffold42686 | FN05724 | *protein DMR6-like oxygenase 2* | Nelumbo nucifera | A0A1U8AFV8 |
|  | ADL | scaffold42686 | FN00351 | *Phytochrome* | Morus notabilis / Cardamine hirsuta / Cardamine nipponica | W9R859 / E9NCZ0 / |
|  | ADL | scaffold42686 | FN23598 | *ovule* | Solanum chacoense | A0A0V0I0B0 |
| QTL*_ADL2_* | ADL | scaffold73277 | FN33349 | *Glutaredoxin family* | Theobroma cacao / Corchorus olitorius | A0A061FT28 / A0A1R3KLQ8 |
| QTL*_BCD2_* | BCD | scaffold13466 | FN06345 |  |  |  |
|  | BCD | scaffold13466 | FN12956 |  |  |  |
|  | BCD | scaffold13466 | FN27674 | *07g29930D* | Brassica napus | A0A078E5K7 |
|  | BCD | scaffold13466 | FN31896 | *BHLH transcription factor* | Glycine soja / Prunus pseudocerasus | A0A0B2RUJ1 / A0A0U2K8U7 |
|  | BCD | scaffold13466 | FN10184 | *Sulfotransferase* | Coffea canephora | A0A068U2D0 |
|  | BCD | scaffold13466 | FN24094 | *Armadillo-type* | Corchorus capsularis | A0A1R3I8Y0 |
|  | BCD | scaffold13466 | FN27076 | *Nodulation H* | Glycine soja | A0A0B2S018 |
|  | BCD | scaffold13466 | FN20264 | *p-loop nucleoside triphosphate hydrolase superfamily* | Medicago truncatula / Theobroma cacao | G7J6A4 / A0A061FCU9 |
|  | BCD | scaffold3696 | FN10275 |  |  |  |
| QTL*_Glc1_* | Glc | scaffold45492 | FN02492 |  |  |  |
|  | Glc | scaffold45492 | FN31533 |  |  |  |
|  | Glc | scaffold45492 | FN23268 |  | Morus notabilis | W9RLP1 |
|  | Glc | scaffold45492 | FN09761 | *ATP-dependent zinc metalloprotease 2, 4 / Peptidase M41* | Gossypium hirsutum / Zostera marina / Macleaya cordata | A0A1U8JXP9 / A0A0K9Q2X3 / A0A200QN61 |
|  | Glc | scaffold45492 | FN09464 |  |  |  |
|  | Glc | scaffold45492 | FN24176 |  |  |  |
|  | Glc | scaffold45492 | FN21907 | *Cysteine desulfurase* | Morus notabilis | W9R1J9 |
|  | Glc | scaffold53823 | FN16068 | *Cytochrom b_5_* | Corchorus capsularis / Morus notabilis | A0A1R3IKW6 / W9R7N2 |
|  | Glc | scaffold53823 | FN28270 |  |  |  |
| QTL*_Glc2_* | Glc | scaffold15962 | FN11160 |  |  |  |
|  | Glc | scaffold15962 | FN25488 |  |  |  |
|  | Glc | scaffold15962 | FN28342 | *Membrane lipo* | Theobroma cacao | A0A061DHT7 |
|  | Glc | scaffold15962 | FN27529 |  |  |  |
|  | Glc | scaffold15962 | FN32391 |  |  |  |
|  | Glc | scaffold15962 | FN20881 |  |  |  |
|  | Glc | scaffold15962 | FN11417 | *Chaperone* | Morus notabilis | W9QNE7 |
|  | Glc | scaffold15962 | FN00219 | *Glycosyltransferase* | Humulus lupulus | W6JNP6 |
|  | Glc | scaffold15962 | FN02004 | *Cysteine-rich receptor kinase 2* | Nicotiana tabacum | A0A1S4D510 |
|  | Glc | scaffold15962 | FN11680 |  |  |  |
|  | Glc | scaffold118257 | FN35255 | *Hevein* | Sambucus nigra | Q944B9 |
| QTL*_Glc3_* | Glc | scaffold137175 | FN29173 | *Phosphoenolpyruvate carboxylase, housekeeping isozyme* | Morus notabilis | W9RZ79 |
| QTL*_GlcA1_* | GlcA | scaffold39478 | FN31042 |  |  |  |
|  | GlcA | scaffold39478 | FN34162 |  |  |  |
|  | GlcA | scaffold39478 | FN35468 | *Homeodomain-like superfamily* | Theobroma cacao | A0A061GGM8 |
|  | GlcA | scaffold39478 | FN26571 |  |  |  |
|  | GlcA | scaffold39478 | FN07437 | *Glutathione S-transferase* | Dimocarpus longan / Theobroma cacao / Cucurbita maxima | I6PBZ4 and I6P8F7 / A0A061EKT0 / Q8H9E5 |
|  | GlcA | scaffold39478 | FN12920 |  |  |  |
|  | GlcA | scaffold90847 | FN34776 |  |  |  |
|  | GlcA | scaffold90847 | FN04787 | *Serine threonine- kinase CBK1* | Morus notabilis | W9QXP3 |
|  | GlcA | scaffold55265 | FN02726 | *p-coumaroyl shikimate 3-hydroxylase* | Garcinia mangostana | A0A068FPP3 |
|  | GlcA | scaffold55265 | FN23690 | *Cytochrome P450* | Atropa belladonna | Q5EKU2 |
|  | GlcA | scaffold79841 | FN35966 | *Sequence-specific DNA binding transcription factors* | Theobroma cacao | A0A061ESQ3 |
| QTL*_GlcA3_* | GlcA | scaffold111279 | FN17464 |  | Morus notabilis | W9SVF1 |
|  | GlcA | scaffold15717 | FN12387 | *Transportin-3* | Cajanus cajan / Glycine soja / Ricinus communis | A0A151QYH4 / A0A0B2SI45 / B9RGS4 |
|  | GlcA | scaffold15717 | FN35911 |  |  |  |
|  | GlcA | scaffold15717 | FN27127 | *MAK16* | Prunus persica | M5VTM6 |
|  | GlcA | scaffold15717 | FN20467 |  |  |  |
|  | GlcA | scaffold15717 | FN03194 |  |  |  |
|  | GlcA | scaffold15717 | FN33744 | *MARCA3 2* | Morus notabilis | W9RK71 |
|  | GlcA | scaffold15717 | FN13972 |  |  |  |
|  | GlcA | scaffold15717 | FN37235 | *SWI/SNF-related matrix-associated actin-dependent regulator of chromatin subfamily A member 3-like 2* | Cucumis melo / Gossypium hirsutum | A0A1S3CG31 / A0A1U8MU20 |
|  | GlcA | scaffold15717 | FN01414 | *ovule* | Solanum chacoense | A0A0V0I8K6 |
|  | GlcA | scaffold15717 | FN10029 | *SNF2-related protein* | Corchorus olitorius | A0A1R3J635 |
|  | GlcA | scaffold15717 | FN00119 |  |  |  |
|  | GlcA | scaffold15717 | FN03562 | *CHR903 / DNA RNA helicase* | Arundo donax / Theobroma cacao | A0A061GWM6 |
|  | GlcA | scaffold15717 | FN23154 | *Nucleosome-remodeling factor subunit BPTF* | Morus notabilis | W9S0X5 |
|  | GlcA | scaffold15717 | FN05826 | *DNA binding* | Ricinus communis | B9SN69 |
|  | GlcA | scaffold15717 | FN29946 | *DUF247 domain* | Medicago truncatula | A0A072TKP8 |
|  | GlcA | scaffold15717 | FN37136 |  |  |  |
|  | GlcA | scaffold15717 | FN00563 | *Glyceraldehyde-3-phosphate dehydrogenase* | Morus notabilis | W9R8N5 |
|  | GlcA | scaffold15717 | FN23221 |  |  |  |
|  | GlcA | scaffold15717 | FN35465 |  | Zostera marina | A0A0K9PMB0 |
|  | GlcA | scaffold15717 | FN23221 | *Truncated A2* | Pisum sativum | E5KXR6 |
|  | GlcA | scaffold15717 | FN02926 | *Peptide chain release factor eRF1 aRF1 / Eukaryotic peptide chain release factor subunit 1-3* | / Vigna radiataCorchorus olitorius / Morus notabilis | A0A1R3JWP9 / W9QM21 / A0A1S3V6B2 |
|  | GlcA | scaffold15717 | FN01757 | *Pentatricopeptide repeat-containing* | Nelumbo nucifera / Ricinus communis / Glycine soja | A0A1U7ZAC9 / B9RGS8 / A0A0B2SHG8 |
|  | GlcA | scaffold15717 | FN03676 | *Acyl-activating enzyme / Acetyl-coenzyme A synthetase* | Cannabis sativa / Gossypium arboreum | H9A8L4 / A0A0B0MP99 |
|  | GlcA | scaffold15717 | FN33671 |  |  |  |
|  | GlcA | scaffold15717 | FN30201 | *PPR domain-containing PPR 2-3* | Cephalotus follicularis / Medicago truncatula | A0A1Q3ANX5 / A0A072V224 |
|  | GlcA | scaffold15717 | FN21194 | *Exportin-1 Importin-beta-like / Xpo1 domain-containing protein* | Macleaya cordata / Cephalotus follicularis | A0A200R3S0 / A0A1Q3CZH5 |
|  | GlcA | scaffold15717 | FN35740 | *Xpo1 domain-containing protein* | Cephalotus follicularis | A0A1Q3CZH5 |
|  | GlcA | scaffold15717 | FN21351 | *Magnesium transporter* | Capsicum annuum / Morus notabilis | A0A1U8FRC9 / W9QUK6 |
|  | GlcA | scaffold15717 | FN37125 |  |  |  |
|  | GlcA | scaffold15717 | FN03079 | *DUF716 domain-containing protein* | Cephalotus follicularis | A0A1Q3C3X3 |
|  | GlcA | scaffold15717 | FN26129 |  |  |  |
|  | GlcA | scaffold15717 | FN21444 | *Syntaxin-22* | Cajanus cajan | A0A151T978 |
|  | GlcA | scaffold15717 | FN19659 |  |  |  |
|  | GlcA | scaffold15717 | FN32535 |  |  |  |
|  | GlcA | scaffold15717 | FN20260 |  |  |  |
|  | GlcA | scaffold15717 | FN10410 |  |  |  |
|  | GlcA | scaffold15717 | FN26657 | *ATP-dependent DNA helicase At3g02060* | Cucumis melo / Nelumbo nucifera | A0A1S3CNQ6 / A0A1U8Q0J7 |
|  | GlcA | scaffold15717 | FN37058 | *Lipid transfer* | Medicago truncatula | A0A072UG85 |
|  | GlcA | scaffold15717 | FN23714 | *Potassium transporter* | Prunus persica / Vitis vinifera | M5X9P3 / F6H416 |
|  | GlcA | scaffold15717 | FN04067 |  |  |  |
|  | GlcA | scaffold15717 | FN07761 | *Eukaryotic translation initiation factor 3 subunit B* | Prunus persica | M5W7G3 |
|  | GlcA | scaffold15717 | FN37096 |  |  |  |
|  | GlcA | scaffold15717 | FN12495 | *Calcium-binding mitochondrial carrier S -1* | Cephalotus follicularis / Corchorus olitorius / Morus notabilis | A0A1Q3CZA7 / A0A1R3JWN1 / W9QUK2 |
|  | GlcA | scaffold15717 | FN01169 | *RNA-binding protein 4* | Anthurium amnicola | A0A1D1YK19 |
|  | GlcA | scaffold15717 | FN27762 |  |  |  |
|  | GlcA | scaffold15717 | FN19953 | *Chalcone synthase* | Cassiope lycopodioides / Cardamine fialae / Humulus lupulus / Nekemias grossedentata | A0A068PX04 / Q1G6S4 / Q94LW8 / R9WQY5 |
|  | GlcA | scaffold15717 | FN08380 | *Stilbene synthase* | Vitis vinifera | Q6BAR5 |
|  | GlcA | scaffold15717 | FN01966 | *Nodulation receptor kinase* | Morus notabilis | W9QM61 |
|  | GlcA | scaffold15717 | FN26409 |  |  |  |
|  | GlcA | scaffold15717 | FN14148 |  |  |  |
|  | GlcA | scaffold15717 | FN20242 | *NADP-dependent alkenal double bond reductase P1* | Morus notabilis | W9S3X7 |
|  | GlcA | scaffold15717 | FN18528 | *Alcohol dehydrogenase family* | Corchorus capsularis / Populus trichocarpa | A0A1R3JYE0 / B9HYF6 |
|  | GlcA | scaffold15717 | FN15087 |  |  |  |
|  | GlcA | scaffold15717 | FN21804 | *LRR receptor-like serine threonine- kinase RPK2* | Morus notabilis | W9QX17 |
|  | GlcA | scaffold15717 | FN16320 |  |  |  |
|  | GlcA | scaffold15717 | FN15641 | *Protein kinase* | Macleaya cordata | A0A200QGU0 |
|  | GlcA | scaffold15717 | FN22343 | *Chromosomal replication initiator* | Gossypium arboreum | A0A0B0N311 |
|  | GlcA | scaffold15717 | FN23542 | *Aminopeptidase* | Arabidopsis thaliana / Zea mays | A0A1P8BGS3 / A0A1D6IUQ0 |
|  | GlcA | scaffold15717 | FN32418 | *Disease resistance* | Theobroma cacao | A0A061EDG0 |
|  | GlcA | scaffold15717 | FN06184 |  |  |  |
|  | GlcA | scaffold15717 | FN30827 | *protein PFC0760c* | Vigna radiata | A0A1S3UZT3 |
|  | GlcA | scaffold15717 | FN07607 |  |  |  |
|  | GlcA | scaffold15717 | FN07300 | *Histone-lysine N-methyltransferase, H3 lysine-9 specific SUVH4* | Cucumis melo / Gossypium hirsutum / Nelumbo nucifera | A0A1S3BN95 / A0A1U8NHJ4 / W9R0J9 / A0A1U7Z9K6 |
|  | GlcA | scaffold15717 | FN32542 |  |  |  |
|  | GlcA | scaffold15717 | FN15157 | *Alpha-mannosidase* | Arabis alpina / Cicer arietinum | A0A087G6Y0 / A0A1S2XQU0 |
| QTL*_KL2_* | KL | scaffold45492 | FN02492 |  |  |  |
|  | KL | scaffold45492 | FN31533 |  |  |  |
|  | KL | scaffold45492 | FN23268 |  | Morus notabilis | W9RLP1 |
|  | KL | scaffold45492 | FN09761 | *ATP-dependent zinc metalloprotease FTSHI 4* | Gossypium hirsutum / Macleaya cordata / Zostera marina | A0A1U8JXP9 / A0A200QN61 / A0A0K9Q2X3 |
|  | KL | scaffold45492 | FN09464 |  |  |  |
|  | KL | scaffold45492 | FN24176 |  |  |  |
|  | KL | scaffold45492 | FN21907 | *Cysteine desulfurase* | Morus notabilis | W9R1J9 |
|  | KL | scaffold69322 | FN21712 |  |  |  |
|  | KL | scaffold69322 | FN08044 | *LIP2* | Arabidopsis thaliana | A0A178UT11 |
|  | KL | scaffold53823 | FN16068 | *Cytochrome b_5_* | Corchorus capsularis / Morus notabilis | A0A1R3IKW6 / W9R7N2 |
|  | KL | scaffold53823 | FN28270 |  |  |  |
|  | KL | scaffold51533 | FN10941 | *protein Trigalactosyldiacylglycerol 5* | Nicotiana tabacum | A0A1S3YTF3 |
|  | KL | scaffold10943 | FN20738 |  |  |  |
|  | KL | scaffold10943 | FN05922 | *Methyltransferase* | Morus notabilis | W9S3D7 |
|  | KL | scaffold63415 | FN06622 |  |  |  |
| QTL*_KL3_* | KL | scaffold6550 | FN26887 | *Caleosin domain-containing* | Cephalotus follicularis | A0A1Q3BFA8 |
|  | KL | scaffold6550 | FN06765 | *Plasmodesmata-located 6* | Theobroma cacao | S1RTU1 |
|  | KL | scaffold6550 | FN28825 |  |  |  |
|  | KL | scaffold6550 | FN11474 |  |  |  |
|  | KL | scaffold6550 | FN13983 | *Plant UBX domain-containing 2* | Theobroma cacao | A0A061E9M4 |
|  | KL | scaffold6550 | FN30281 |  |  |  |
|  | KL | scaffold6550 | FN16129 | *Med23 domain-containing* | Cephalotus follicularis | A0A1Q3BFG8 |
|  | KL | scaffold6550 | FN17693 | *Mediator of RNA polymerase II transcription subunit 23* | Arabidopsis thaliana / Cajanus cajan / Cephalotus follicularis / Gossypium hirsutum / Nicotiana tabacum / Theobroma cacao / Vigna radiata | A0A1P8AUG4 / A0A151T8F0 / A0A1Q3BFG8 / A0A1U8JS22 and A0A1U8I8P8 / A0A1S3X307 / A0A061E9L9 / A0A1S3VR89 |
|  | KL | scaffold6550 | FN11795 | *Glucan endo-1,3-beta-glucosidase 3 / F-box protein SKIP28* | Morus notabilis / Cicer arietinum and Vigna radiata | W9SPC5 / A0A1S2YDV5 and A0A1S3UV78 |
|  | KL | scaffold6550 | FN24577 |  |  |  |
|  | KL | scaffold6550 | FN34858 |  |  |  |
|  | KL | scaffold6550 | FN14704 |  | Pyrus betulifolia |  |
|  | KL | scaffold12000 | FN36163 |  |  |  |
|  | KL | scaffold12000 | FN06609 |  |  |  |
|  | KL | scaffold12000 | FN17560 |  |  |  |
|  | KL | scaffold12000 | FN20295 | *recQ-mediated genome instability protein 1 / ovule* | Cucumis melo / Solanum chacoense | A0A1S3CLI7 / A0A0V0IM89 |
|  | KL | scaffold12000 | FN34987 | *RBR-type E3 ubiquitin transferase* | Cephalotus follicularis | A0A1Q3BGU2 |
|  | KL | scaffold12000 | FN02338 | *HDR (4-hydroxy-3-methylbut-2-enyl diphosphate reductase) / ResB domain-containing protein* | Cannabis sativa / Cephalotus follicularis / Macleaya cordata | A0A1V0QSH9 / A0A1Q3BGI1 / A0A1V0QSH9 |
| QTL*_Man1_* | Man | scaffold47229 | FN29754 |  |  |  |
|  | Man | scaffold47229 | FN24336 |  |  |  |
|  | Man | scaffold47229 | FN23575 | *Ferric-chelate reductase 1* | Morus notabilis | W9R800 |
|  | Man | scaffold47229 | FN22376 | *AT1G08220* | Francoa sonchifolia | A0A0G4ALR4 |
|  | Man | scaffold47229 | FN00845 |  |  |  |
|  | Man | scaffold47229 | FN26085 | *Lysosomal Pro-X carboxypeptidase* | Ricinus communis | B9SCN3 |
|  | Man | scaffold47229 | FN22206 | *Vacuolar sorting-associated 41* | Cephalotus follicularis / Morus notabilis / Theobroma cacao | A0A1Q3CCQ5 / W9RC41 / A0A061GVY4 |
|  | Man | scaffold2731 | FN19737 | *Helicase MAGATAMA 3* | Corchorus olitorius / Macleaya cordata | A0A1R3JHE1 / A0A200PY02 |
|  | Man | scaffold2731 | FN31670 |  |  |  |
|  | Man | scaffold2731 | FN17565 | *AT2G19120-like protein* | Capsella grandiflora | G3LLC8 |
|  | Man | scaffold2731 | FN02925 | *ATP-dependent helicase* | Cucumis melo | A0A1S3B945 |
|  | Man | scaffold2731 | FN09557 |  |  |  |
|  | Man | scaffold34580 | FN14046 | *Pentatricopeptide repeat-containing* | Cicer arietinum / Gossypium hirsutum / Nicotiana sylvestris / Ricinus communis | A0A1S3EF11 / A0A1U8LE64 / A0A1U7YJU2 / B9T4W2 |
|  | Man | scaffold34580 | FN02656 | *Transmembrane 220* | Anthurium amnicola | A0A1D1YV08 |
|  | Man | scaffold34580 | FN34305 | *LRR receptor-like serine threonine- kinase MEE39* | Morus notabilis | W9R9T5 |
|  | Man | scaffold34580 | FN33995 | *Kinase family peptidoglycan-binding domain-containing* | Theobroma cacao | A0A061DFY3 |
|  | Man | scaffold34580 | FN36128 | *Cysteine-rich receptor kinase 19* | Morus notabilis | W9RYJ2 |
|  | Man | scaffold34580 | FN16350 | *Glucose-1-phosphate adenylyltransferase* | Morus notabilis | W9R328 |
|  | Man | scaffold90847 | FN34776 |  |  |  |
|  | Man | scaffold90847 | FN04787 | *Serine threonine- kinase CBK1* | Morus notabilis | W9QXP3 |
| QTL*_Man2_* | Man | C32103569 | FN15574 | *Arginine biosynthesis bifunctional* | Gossypium arboreum | A0A0B0MPM1 |
| QTL*_Xyl1_* | Xyl | scaffold55265 | FN02726 | *p-coumaroyl shikimate 3-hydroxylase* | Garcinia mangostana | A0A068FPP3 |
|  | Xyl | scaffold55265 | FN23690 | *Cytochrome P450* | Atropa belladonna | Q5EKU2 |

QTL region= name given to the common QTL region across locations; Trait= fibre quality trait of interest; Scaffold= genomic scaffold with markers associated to the QTL region; Transcript= transcript of the transcriptome of hemp cultivar ‘Finola’ homologous to a sequence of the scaffold; Homologous gene= gene homologous to the sequence of the transcript identified or predicted in other species; Species= species where the homologous gene was identified; UniProt code= protein code of the homologous gene. Scaffolds composing QTL regions from **Table 5** missing in **Supplementary Table 1** showed no transcripts. ADL= Acid Detergent Lignin, BCD= Bast fibre content after decortication, Glc= Glucose, GlcA= Glucuronic acid, KL= Klason lignin, Man= Mannose and Xyl= Xylose.

**Supplementary Table 6.** Arabidopsis homologues of the candidate genes detected by blasting the sequences [using Blast+ software (Camacho et al., 2009)] to the hemp annotated transcriptome BioProject PRJNA435671 (Behr et al., 2019).

| Candidate gene (Transcript) | Description | Transcripts from Transcriptome Behr et al., 2019 | Arabidopsis homologues codes |
| --- | --- | --- | --- |
| FN14269 | Polygalacturonase | contig_25296 | BAC42276.1; BAB01843.1; AEE77185.1; NP_189293.1; CAA66811.1 |
| FN26643 | 2-phytyl-1,4-beta-naphthoquinone methyltransferase | contig_15271; contig_8121; contig_8719; contig_19519 | NP_173750.3; OAP18287.1; BAH19903.1; Q3ED65.2; AEE30377.1 |
| FN00219 | Glycosyltransferase | contig_35719; contig_34971 | AAM13356.1; AAL32657.1 |
| FN29173 | Phosphoenolpyruvate carboxylase, housekeeping isozyme | contig_2810 | AAF69546.1; CAD58725.1; OAP17695.1; AAL09748.1; AEE32921.1; NP_175738.1; NP_001031178.1; Q9MAH0.1; AAN18213.1; NP_001031179.1; AEE32923.1; AEE32922.1 |
| FN02726 | p-coumaroyl shikimate 3-hydroxylase | - | - |
| FN00563 | Glyceraldehyde-3-phosphate dehydrogenase | contig_3629 | AAL24215.1; AAL25556.1; OAP05662.1; BAB01730.1; AAL91645.1; AAM98317.1; AAL16200.1; AEE77191.1 ;NP_566796.2; P25856.3 |
| FN03676 | Acyl-activating enzyme / Acetyl-coenzyme A synthetase | contig_5964 | OAO91408.1 |
| FN19953 | Chalcone synthase | contig_24810 | CDU43901.1; AAM65314.1; CDU43904.1 |
| FN08380 | Stilbene synthase | - | - |
| FN15157 | Alpha-mannosidase | contig_244; contig_1173 | Q8LPJ3.1; AAN15620.1;AED91968.1;AED91969.1;AAM20555.1;NP_196902.2;NP_851037.2 |
| FN05922 | Methyltransferase | contig_4404 | NP_196644.1; AAO24556.1; CAB96838.1; BAF00142.1; AED91601.1 |
| FN11795 | Glucan endo-1,3-beta-glucosidase 3 / F-box protein SKIP28 | contig_7249 | Q9ZU91.2; NP_565269.1; AAD12708.2; AEC05475.1; AAM62724.1 |
| FN16350 | Glucose-1-phosphate adenylyltransferase | contig_22044 | AAK43880.1; AAB58475.1; CAA51779.2; NP_197423.1; AED92672.1; AAP68323.1; P55229.3 |
| FN02726 | p-coumaroyl shikimate 3-hydroxylase | - | - |

Candidate gene= transcript of hemp cultivar ‘Finola’ (transcriptome assembly; Finola) located in a QTL across locations, annotated for a putative function in cell wall metabolism and/or fibre quality (Blast2go; gene description, relevant research papers); Description= description of hemp transcript, as found by Blast2Go annotation, based on homology to sequence from GenBank with known or predicted functions; Transcripts from Transcriptome Behr et al., 2019= transcripts of hemp cultivar ‘Santhica 27’ that blasted with transcripts of hemp cultivar ‘Finola’; Arabidopsis homologues codes= codes of arabidopsis sequences homologues to the candidate genes belonging to the annotations of the transcripts from Behr et al., 2019.

**References Supplementary data**

Berh, M., Lutts, S., Hausman, J.F., Sergeant, K., Legay, S., Guerriero, G. (2019). *De novo* transcriptome assembly of textile hemp from datasets on hypocotyls and adult plants. Data Brief 27, 104790. doi: 10.1016/j.dib.2019.104790.

Petit, J., Salentijn, E.M.J., Paulo, M.J., Thourminot, C., Van Dinter, B.J., Magagnini, G., Gusovius, H.J., Tang, K., Amaducci, S., Wang, S., Uhrlaub, B., Mussig, J., and Trindade, L.M. (2020). Genetic variability of morphological, flowering and biomass quality traits in hemp (*Cannabis sativa* L.). *Front. Plant Sci.* 10. doi: 10.3389/fpls.2020.00102.

Camacho, C., Coulouris, G., Avagyan, V., Ma, N., Papadopoulos, J., Bealer, K., Madden, T.L. (2009). BLAST+: architecture and applications. *BMC Bioinformatics* doi: 10.1186/1471-2105-10-421
